# Effects of Plectin Depletion on Keratin Network Dynamics and Organization

**DOI:** 10.1371/journal.pone.0149106

**Published:** 2016-03-23

**Authors:** Marcin Moch, Reinhard Windoffer, Nicole Schwarz, Raphaela Pohl, Andreas Omenzetter, Uwe Schnakenberg, Fabian Herb, Kraisorn Chaisaowong, Dorit Merhof, Lena Ramms, Gloria Fabris, Bernd Hoffmann, Rudolf Merkel, Rudolf E. Leube

**Affiliations:** 1 Institute of Molecular and Cellular Anatomy, RWTH Aachen University, Aachen, Germany; 2 Institute of Materials in Electrical Engineering 1, RWTH Aachen University, Aachen, Germany; 3 Institute of Imaging & Computer Vision, RWTH Aachen University, Aachen, Germany; 4 Institute of Complex Systems, Forschungszentrum Jülich, Jülich, Germany; Sanford Burnham Medical Research Institute, UNITED STATES

## Abstract

The keratin intermediate filament cytoskeleton protects epithelial cells against various types of stress and is involved in fundamental cellular processes such as signaling, differentiation and organelle trafficking. These functions rely on the cell type-specific arrangement and plasticity of the keratin system. It has been suggested that these properties are regulated by a complex cycle of assembly and disassembly. The exact mechanisms responsible for the underlying molecular processes, however, have not been clarified. Accumulating evidence implicates the cytolinker plectin in various aspects of the keratin cycle, i.e., by acting as a stabilizing anchor at hemidesmosomal adhesion sites and the nucleus, by affecting keratin bundling and branching and by linkage of keratins to actin filament and microtubule dynamics. In the present study we tested these hypotheses. To this end, plectin was downregulated by shRNA in vulvar carcinoma-derived A431 cells. As expected, integrin β4- and BPAG-1-positive hemidesmosomal structures were strongly reduced and cytosolic actin stress fibers were increased. In addition, integrins α3 and β1 were reduced. The experiments furthermore showed that loss of plectin led to a reduction in keratin filament branch length but did not alter overall mechanical properties as assessed by indentation analyses using atomic force microscopy and by displacement analyses of cytoplasmic superparamagnetic beads using magnetic tweezers. An increase in keratin movement was observed in plectin-depleted cells as was the case in control cells lacking hemidesmosome-like structures. Yet, keratin turnover was not significantly affected. We conclude that plectin alone is not needed for keratin assembly and disassembly and that other mechanisms exist to guarantee proper keratin cycling under steady state conditions in cultured single cells.

## Introduction

Keratins are main components of the epithelial cytoskeleton which are anchored to desmosomes at cell-cell junctions and to hemidesmosomes at the cell-extracellular matrix interface [1, 2]. The 8–12 nm-thick keratin filaments are composed of equimolar amounts of type I and type II keratin polypeptides [3]. Depending on keratin isotype and cellular background keratins are arranged in different types of complex networks [4].

The initial image of the keratin cytoskeleton as a highly rigid and static scaffolding has been substituted by the concept of a mechanically resilient system with a very high degree of intrinsic dynamics and responsive plasticity to act as an efficient buffering system for the many different stresses imposed on epithelial tissues [2, 5]. The rapid disassembly of the keratin cytoskeleton within minutes in dividing cells during prophase and its subsequent re-assembly at the end of mitosis as well as the polarized keratin network assembly in the leading edge of migrating cells are just two examples of exceptional keratin network plasticity [6, 7]. Even at equilibrium, the keratin cytoskeleton has been shown to be subject to a continuous cycle of assembly and disassembly [6, 8]. In cultured cells, this keratin cycle is characterized by nucleation of keratin particles in the cell periphery, followed by elongation and end-on integration of filamentous particles into the peripheral network. These processes are coupled to actin-dependent inward transport, which continues as the filaments within the network mature into thicker bundles. Some of these filaments are stabilized through anchorage to the nucleus, while others disassemble into rapidly diffusible subunits, which are reutilized for another round of assembly and disassembly. The keratin cycle is regulated by EGF signaling [9] presumably through posttranslational modifications such as phosphorylation, sumoylation and cysteine cross-linking [10, 11] and by interaction with other cytoskeletal components through proteinaceous cross linkers [12, 13]. Among the latter, plectin is certainly the most conspicuous candidate [6, 13, 14]. It has been shown to attach keratins to hemidesmosomal integrin β4 [15–19] and to the nuclear envelope through nesprin 3 [20]. It has also been implicated in keratin bundling [21] and keratin branching [22]. Given the observed nucleating function of plectin splice variant 1f for vimentin intermediate filaments at focal adhesions [23], a similar function could be envisioned for the epithelial counterparts. Finally, based on its binding capacity for actin filaments and keratins [24, 25], plectin could link inward transport of keratins to actin retrograde flow in a piggyback-fashion in the cell periphery.

To examine these multiple functions of plectin for keratin network organization, plectin expression was downregulated by shRNA in vulvar carcinoma derived A431 cells, which express keratin 13-EGFP. Quantitative analyses of keratin network morphology, cell mechanics and keratin dynamics were performed. We observe that plectin depletion decreases the branching of keratin networks and regulates the expression of multiple integrins. Yet, loss of plectin only increases the motility of keratins, which can be explained by the absence of hemidesmosome-like structures, but does not affect overall keratin turnover and global cell stiffness.

## Materials and Methods

### Cell Culture

A431 vulvar carcinoma derived cells (wild type) and subclone AK13-1 were described in [26]. The wild type cells and all derived subclones were grown in DMEM without phenol red supplemented with GlutaMAX™ (Life technologies) and 10% (v/v) fetal calf serum (FCS; Invitrogen). For passaging, cells were washed with PBS without Ca^2+^/Mg^2+^ (Biochrom) supplemented with 0.02% (w/v) EDTA (Sigma-Aldrich) and trypsinized in the same solution containing 0.25% (w/v) trypsin (Biochrom) for ≈ 1 minute (min). The cells were passaged two times per week and all experiments were performed within 15 passages. Immortalized human HaCaT keratinocytes were kindly provided by Dr. Petra Boukamp [27]. They were grown under the same conditions as vulvar carcinoma-derived cells and passaged with the same reagents once a week, i.e., one day after reaching 100% confluence. Therefore, cells were incubated for 5 min in PBS/EDTA followed by a 5 min treatment with trypsin/PBS/EDTA at 37°C. Prior to experiments vulvar carcinoma-derived cells were grown on laminin 332-rich matrices derived from 804G cells [9], with the exception of magnetic tweezers experiments where uncoated dishes were used. HaCaT cells were always grown on uncoated surfaces.

### Cloning of Plectin shRNA

The lentiviral vector pLVTHM-CFP was provided by Dr. Anne Kölsch and was used as a backbone for all shRNA constructs. It was generated by restriction of pLVTHM [28] (Addgene plasmid #12247) with *Pme*I/*Spe*I and insertion of an ECFP-encoding fragment with complementary restriction sites. The insert was generated by PCR from pECFP-N1 (Clontech Laboratories) using primers 5'-CGA TGT TTA AAC CTC GGA CTC AGA TCT CGA G-3' and 5'-GCA CTA GTC AAA TGT GGT ATG GCT GAT TAT G-3'. The shRNA sequences to be inserted were ordered as lyophilized primer nucleotides (Eurofins MWG). Plectin shRNA: 5'-CGC GTC CCC **GCC AGT ACA TCA AGT TCA TCA** TTC AAG AGA TGA **TGA ACT TGA TGT ACT GGC** TTT TTG GAA AT-3' and 5'-CGA TTT CCA AAA A**GC CAG TAC ATC AAG TTC ATC A**TC TCT TGA ATG A**TG AAC TTG ATG TAC TGG** CGG GGA-3' (targeting sequences are marked in bold). Scramble control shRNA: 5'-CGC GTC CCC C**CG TCA CAT CAA TTG CCG T**TT CAA GAG A**AC GGC AAT TGA TGT GAC GG**T TTT TGG AAA T-3' and 5'-ATT TCC AAA A**AC CGT CAC ATC AAT TGC CGT** TCT CTT GAA **ACG GCA ATT GAT GTG AC**G GGG. For hybridization complementary oligonucleotides were dissolved at 5 pmol μl^-1^ in 40 μl double distilled H_2_O before heating for 2 minutes at 95°C following hybridization at room temperature for 2 hours (h). The hybridized linkers were phosphorylated by T4 polynucleotide kinase (Thermo Fisher Scientific) and were inserted into pLVTHM-CFP using *Cla*I and *Mlu*I restriction sites. The generated plasmids were checked by sequencing (Eurofins MWG).

### Immunoblot Analyses

Immunoblot analyses were performed as described in [9] with the following modifications. Proteins were transferred onto PVDF membranes over night at 37 V except for keratin 17 immunoblots which were done at 100 V for 100 min. Membranes with bound proteins were blocked for 1 h in 10% (v/v) Roti®-Block (Carl Roth) and incubated with primary antibodies in buffer [50 mM Tris, 130 mM NaCl, 0.1% (v/v) Tween 20, pH 7.6] containing 1% or 2% or 10% (v/v) Roti®-Block. The same buffer with 1% (v/v) Roti®-Block was used for secondary antibody incubation. Bound antibodies were detected with AceGlow™ Chemiluminescence Substrate (Peqlab) on a Fusion-Solo.WL.4M with Fusion-Capt Advance Software 16.06 (Vilber Lourmat). Protein mass was determined with the ProSieve QuadColor Protein Marker (Lonza). Unprocessed immunoblots and protein transfer controls are included as S1 Files.

### Immunocytochemistry

Cells were either grown in glass-bottom dishes (35 mm Petri dish with 14 mm glass surface, #1.5, MatTek) or on glass cover slips (d = 24 mm, #1.5, Gerhard Menzel). They were washed shortly with PBS at 37°C and fixed for 3 min in methanol followed by a 30 s immersion in acetone at -22°C. In case of actin staining with Alexa Fluor® 555-conjugated phalloidin (Invitrogen) cells were fixed for 10 min in 4% (w/v) paraformaldehyde (Merck) in PBS (pH 7.2–7.4; adjusted with NaOH at ≈ 70°C) at room temperature and permeabilized with 0.2% (v/v) Triton™ X-100 (Sigma-Aldrich) in PBS for 3 min. Fixed cells were washed in PBS and blocked for 1 h in PBS containing 5% (w/v) bovine serum albumin (BSA; Sigma-Aldrich). Thereafter, cells were incubated with primary antibodies for 1 h, washed with PBS, and incubated with secondary antibodies for 40 min [antibodies were dissolved in PBS containing 1% (w/v) BSA]. Before mounting with Mowiol (Carl Roth) on glass slides (76x26 mm; R. Langenbrinc) cells were washed with PBS and mono-distilled H_2_O. Samples were dried over night at 4°C and stored at the same temperature until usage (within two weeks).

### Antibodies

Primary monoclonal murine antibodies against integrins α2 (dilution: 1:500; clone: 2/CD49b; Lot No.: 81934), α3 (1:300; 42/CD49c; 78973), α5 (1:500; 1/CD49e; 81386), β1 (1:500; 18/CD29; 78008), β4 (1:500 for immunoblot; 7/CD104; 78968) were from BD Bioscience, against GAPDH (1:2000; 9484; GR9686-1) from Abcam, against keratin 13 (1:300; Ks 13.1) from Progen Biotechnik, against BPAG-1 (1:100; 279; 141125) from Cosmo Bio Corporation, against plectin/HD1 (1:200) from Katsushi Owaribe [29], and against keratins 8/18 (1:2500) from Bishr Omary [30]. Primary monoclonal rabbit antibodies against integrin β5 (1:200 for immunocytochemistry and 1:1000 for immunoblot; D24A5; 0001) were from Cell Signalling Technology. Primary TROMA-I monoclonal rat antibodies against keratin 8 (1:10) were described in [31] and were produced in cells from The Developmental Studies Hybridoma Bank. Primary polyclonal rat antibodies against integrin β4 (1:200 for immunocytochemistry; 439-9B; 45416) were from BD Pharmingen, and against β-actin (1:2000; Lot No.: 057K4803) from Sigma-Aldrich. Primary polyclonal guinea pig antibodies against keratin 5 (1:5000; Lot No.: 302041) were from Progen Biotechnik. For information on secondary antibodies see antibodies.pdf included in S1 Files.

### Microscopy

Recordings were performed at 16 bit signal resolution with a laser scanning confocal microscope (LSM 710 DUO) equipped with an oil immersion objective (63×/1.40-N.A. DIC M27) and 405/488/543/633 nm lasers. For live-cell imaging, the microscope was pre-warmed to 37°C in a climate chamber and focus was automatically corrected every 2 seconds with the Definite Focus system (all from Carl Zeiss). Living cells were imaged in glass-bottom dishes in HEPES-buffered DMEM without phenol red supplemented with GlutaMAX (Life Technologies) either with 10% FCS or without FCS. The recording of keratin 13-EGFP in single cells was performed at the cell bottom by unidirectional scanning of an 79.3 μm × 79.3 μm area with a 488 nm argon laser (2.5% laser power; pixel dwell time: 12.6 μs; pinhole: 2 airy units; light control potentiometer set to standby; 488/543 dichroic beam splitter). The emitted light was recorded with a photomultiplier tube at 480–599 nm at a resolution of 1024 × 1024 pixels every 30 s. Immunostained cells were imaged at settings that allowed reliable emission spectra separation.

### Determination of Keratin Branch Length

A pipeline of algorithms was applied to confocal images of HK13-EGFP in AK13-1 subclones resulting in a geometrical representation which can be used for morphology analysis e.g. branch length determination of the filaments. First, a basic Gaussian filter was used for noise reduction. An optimally oriented flux filter was then applied [32]. It enhances curvilinear structures, notably filaments, in the image thereby further suppressing noise. To convert the output of the filter into a binary image, a basic thresholding method was applied. A centerline extraction algorithm then reduced all structures in the binary image to a width of one voxel. A 3D line reconstruction method was then used to transform the centerline image into a geometrical representation. In some cases, the centerline extraction algorithm leaves branch points consociating to more than one voxel. These are not considered as one branch point by the line reconstruction. Therefore, these branch points were merged into one point in a subsequent step to preserve the important connectivity information at these points. At the end, a geometric low pass filter was applied to smooth out aliasing effects caused by the discrete imaging process. Results of single measurements are included in S1 Files.

### Atomic Force Measurements

For atomic force measurements (AFM), 60 000 cells were seeded on laminin 332-rich matrices and kept in serum-free medium 24 h before each experiment. Immediately before measurements, cells were immersed in freshly prepared serum-free medium. AFM force spectroscopy on living cells was realized using a Nanowizard Life Science instrument (JPK) combined with an inverted optical microscope (Axiovert 200, Zeiss) for sample observation. Probing of cell elasticity was performed by using tipless cantilevers (f_0_ = 7 kHz, k = 0.04 N/m; Nanoworld Arrow TL1Au with Ti/ Au back tip coating) equipped with a silica microsphere of a nominal radius of 2.25 μm (G. Kisker GbR, PSI-5.0, surface plain) as described previously [33]. For indentation experiments, three force curves were recorded for each position with a cantilever speed of 1.5 μm/s and a force setpoint of 1.5 nN. All indentation measurements were performed above the nucleus. Results of single measurements are included in S1 Files.

### Force-Distance Curve Analysis

Force-distance curves (F-D curves) were fitted using a standard power law function *F = Aδ*^*b*^, with apparent stiffness *A* and exponent *b* as free fit parameters. The general power law function can be regarded as a generalization of the usual Hertz model; for details see [33]. Fitting all F-D curves, we obtained mean exponent values of 1.80 ±0.27 (SD). The exponent was therefore fixed to a value of 1.8 and apparent stiffness (*A*_*1*.*8*_) was fitted for all F-D curves. The parameter-free two-sided Mann-Whitney-Wilcoxon test was used to analyze differences in the distributions of resulting apparent stiffness A_1.8_. Results of single measurements are included in S1 Files.

### Magnetic Tweezers Data Analysis

A magnetic tweezers setup described in [33] was calibrated and used for measurements of cytoplasmic viscoelastic properties. In short, M-270 superparamagnetic Dynabeads (Invitrogen) were incorporated into the cell’s cytoplasm using the fusion reagent Fuse-It-Beads Ibidi). In each experiment, a magnetic needle kept at a distance of 40–60 μm from a single cell was used to apply, every 10 s, repeated rectangular pulses of 5 s duration to the paramagnetic bead. The positions of the beads and needle tips were recorded at a frequency of 50 frames/s by an EOS 650D/Rebel T4i digital single-lens reflex (DSLR) camera (Canon), and were tracked via implemented algorithms of Adobe After Effect CS4 (Adobe Systems) and Fiji software (freely available at http://fiji.sc/Fiji).

Single bead displacement peaks were normalized with the magnetic force applied at every time frame, and creep response curves were fit to the simple power law (equation 1):

$$J\left(t\right)=J_{0}\;{\left(\frac{t}{t_{0}}\right)}^{\beta }$$

where $\frac{1}{J_{0}}=K_{0}$ is equivalent to a shear modulus, and *β* characterizes the viscoelastic properties of the cytoplasm [34]. A total of 67 peaks (12 cells) were analyzed for the wild type, 107 peaks (19 cells) for the scramble control, and 117 peaks (20 cells) for the shPlectin cells. Results of single measurements are included in S1 Files.

## Results

### Efficient Downregulation of Plectin in Cultured Cells Results in Loss of Hemidesmosome-Like Structures, Altered Integrin Expression and Slightly Changed Keratin and Actin Network Morphology

**Fig 1:**
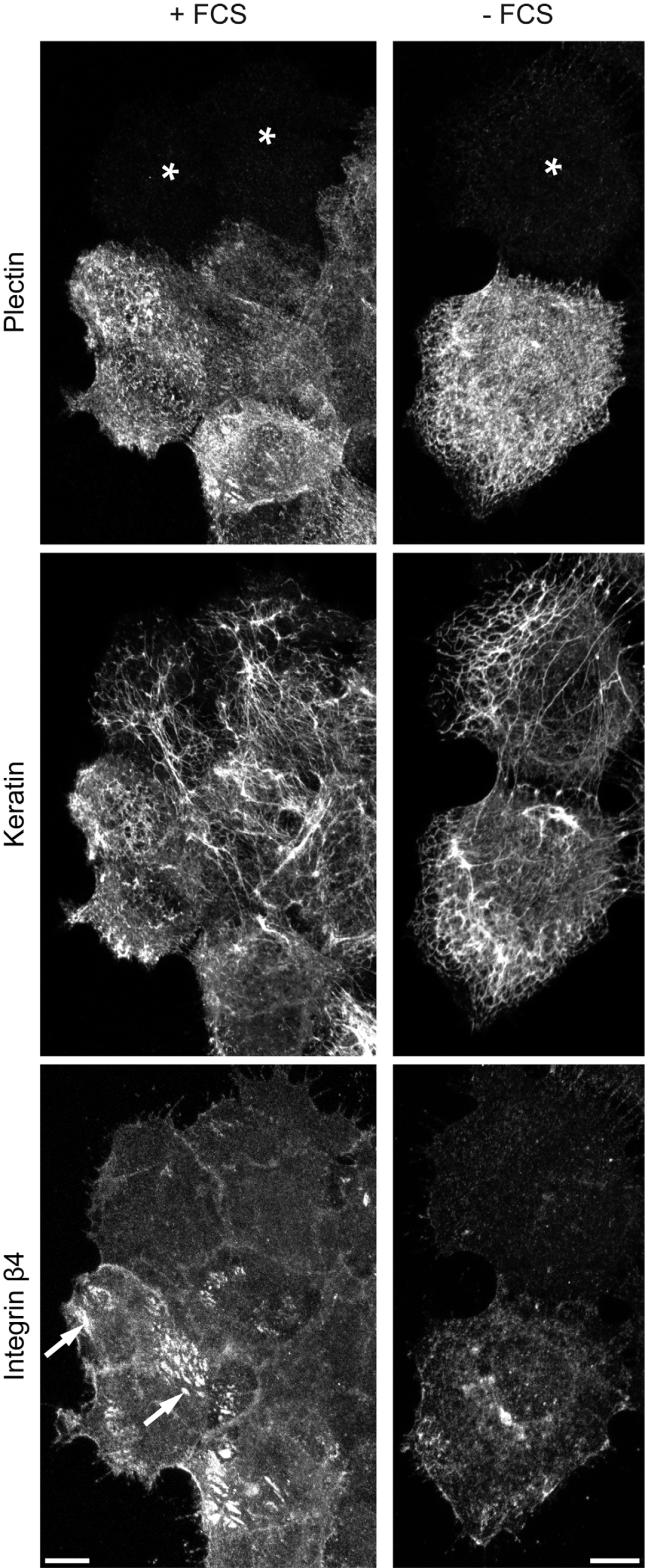
Plectin knock down prevents formation of integrin β4 clusters and slightly affects overall keratin network morphology in A431 cells. Vulvar carcinoma derived A431 cells were plated on laminin 332-rich matrix 48 h prior to methanol/acetone fixation. The images show triple immunofluorescence staining of plectin (guinea pig antibodies), integrin β4, and pan-keratin. They demonstrate the efficient down regulation of plectin by shRNA in cells marked with an asterisk in comparison to non-transfected neighboring cells. They also show that hemidesmosomal integrin β4 clusters (arrows) are formed in the presence of FCS in contrast to plectin-free cells and serum-starved cells (+FCS vs. -FCS). Note the less dense and more compact keratin filament networks in plectin-depleted cells. All images are maximum intensity projections from confocal sectioning of complete cells. Bars, 10 μm.

Plectin shRNA was generated against nucleotides 4573–4593 in exon 31 of human plectin 1 (NCBI reference sequence NM_201380) that are shared by all known plectin isoforms. The selected scramble shRNA control is not represented in the human genome. For transduction, a lentiviral system was chosen that additionally encodes ECFP. The effect of plectin downregulation by shRNA was first tested by transient transduction of vulvar carcinoma-derived A431 cells (Fig 1). Plectin was clearly detectable in non-transduced cells, where it co-localized in part with keratin filaments and integrin β4-clusters. These structures were previously described to be related to type I hemidesmosomes. They contain besides α6β4 integrin and HD1/plectin, BPAG-1 and BPAG-2 [35, 36]. Plectin was almost undetectable in transduced cells two weeks after infection (asterisks). Depletion of plectin led to loss of hemidesmosome-like integrin β4-positive structures as expected [18, 37]. Integrin β4-staining was instead more diffuse at cell-cell borders and appeared to be less strong (Fig 1). When cells were cultivated in the absence of FCS, integrin β4-staining was altered from the patchy hemidesmosome-like pattern to a less pronounced diffuse and/or multipunctate distribution indicating loss of hemidesmosome-like structures. Reduction of integrin β4-immunostaining could be an overestimate because the used methanol fixation may have removed cytoplasmic integrin β4. The keratin networks in plectin-depleted cells appeared to be less dense and contained thicker filaments. This was especially noticeable in flat cells at the edges of cell groups. Additionally, the effects of plectin shRNA were examined in HaCaT keratinocytes. Plectin-immunostaining showed a more pronounced hemidesmosomal localization and less filamentous distribution (S1A and S1B Fig). Plectin shRNA again efficiently reduced plectin levels resulting in loss of integrin β4-positive hemidesmosome-like structures and barely visible changes in keratin network organization (S1A Fig).

**Fig 2:**
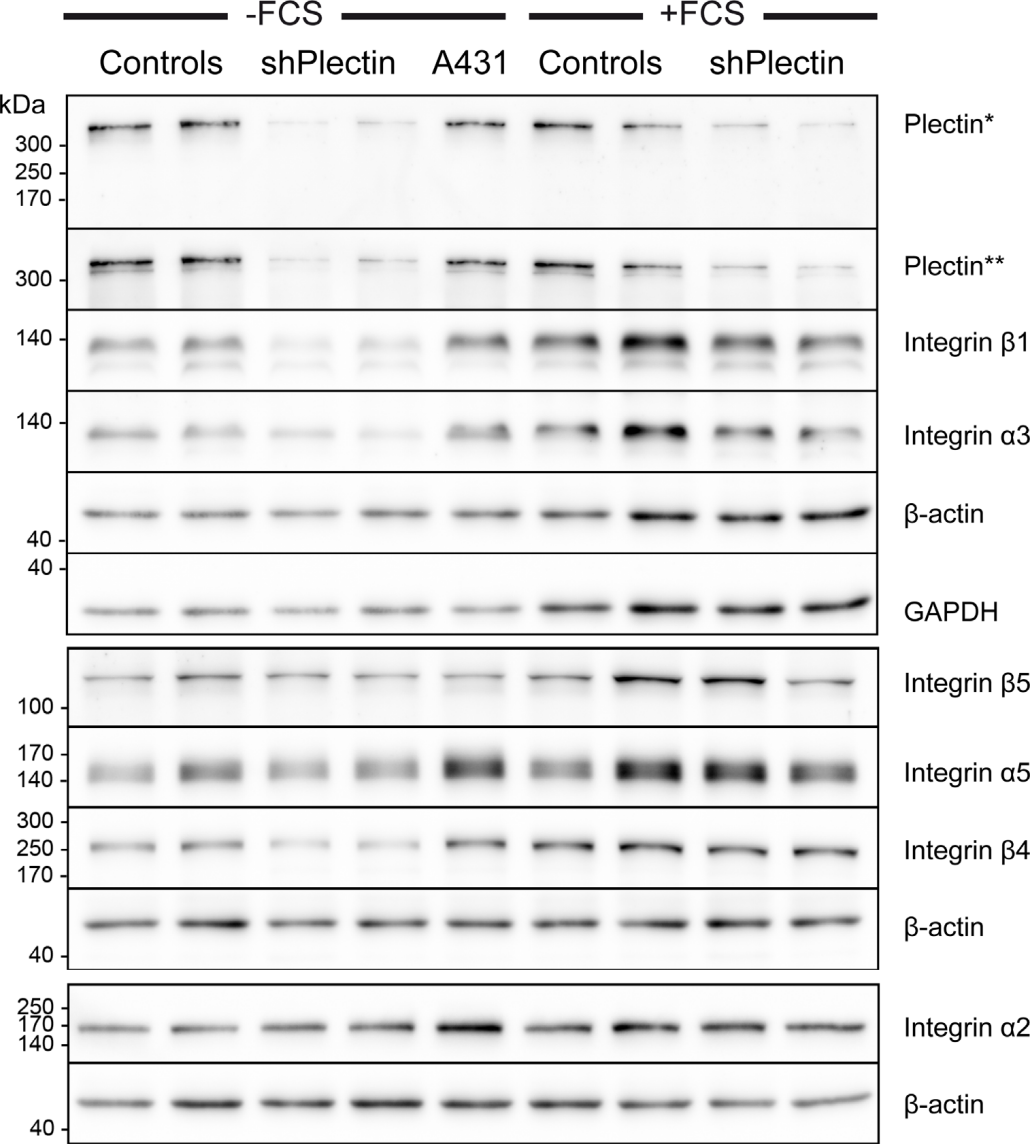
Immunoblots detect efficient reduction of plectin and complex alterations of integrin expression in A431 clones stably transfected with plectin shRNAs. Complete cell lysates were obtained from AK13-1-derived subclones that were either stably transfected with scramble shRNA (controls) or plectin shRNA (shPlectin). For comparison, cell lysates from non-transfected A431 cells were prepared. The cells had been cultivated on laminin 332-rich matrices for 48 h either without (left) or with FCS (right). Proteins were separated by 8% SDS-PAGE. Note the efficient down regulation of plectin by 80–90% in both plectin shRNA clones as detected by two different antibodies (*guinea pig, **mouse). In addition, integrins β1 and α3 are also noticeably reduced in plectin-deficient cells. Integrin β4 is only reduced in serum-starved cells upon plectin depletion. The changes in expression levels of other integrins are apparently not linked to plectin expression. Note also, that integrin expression is increased in the presence of serum.

shRNA-producing subclones were generated from A431 cell clone AK13-1, which stably expresses keratin 13-EGFP [26]. For further detailed analyses, the two clones with the strongest plectin depletion and two random clones producing scramble shRNA were selected. Plectin was downregulated in both plectin shRNA clones by ≈ 80–90% as determined by immunoblot analysis (n = 3) using two different antibodies (Fig 2). Furthermore, the protein levels of integrin α3 and β1 were decreased upon plectin-depletion. The amount of integrin β4 was also decreased, but only in cells cultivated without FCS. Otherwise, the protein levels of β-actin, GAPDH, integrin α2, and integrin β5 were not affected by plectin depletion. This was also the case for keratins 5, 8, 13, 17, and 18 (S2 Fig).

**Fig 3:**
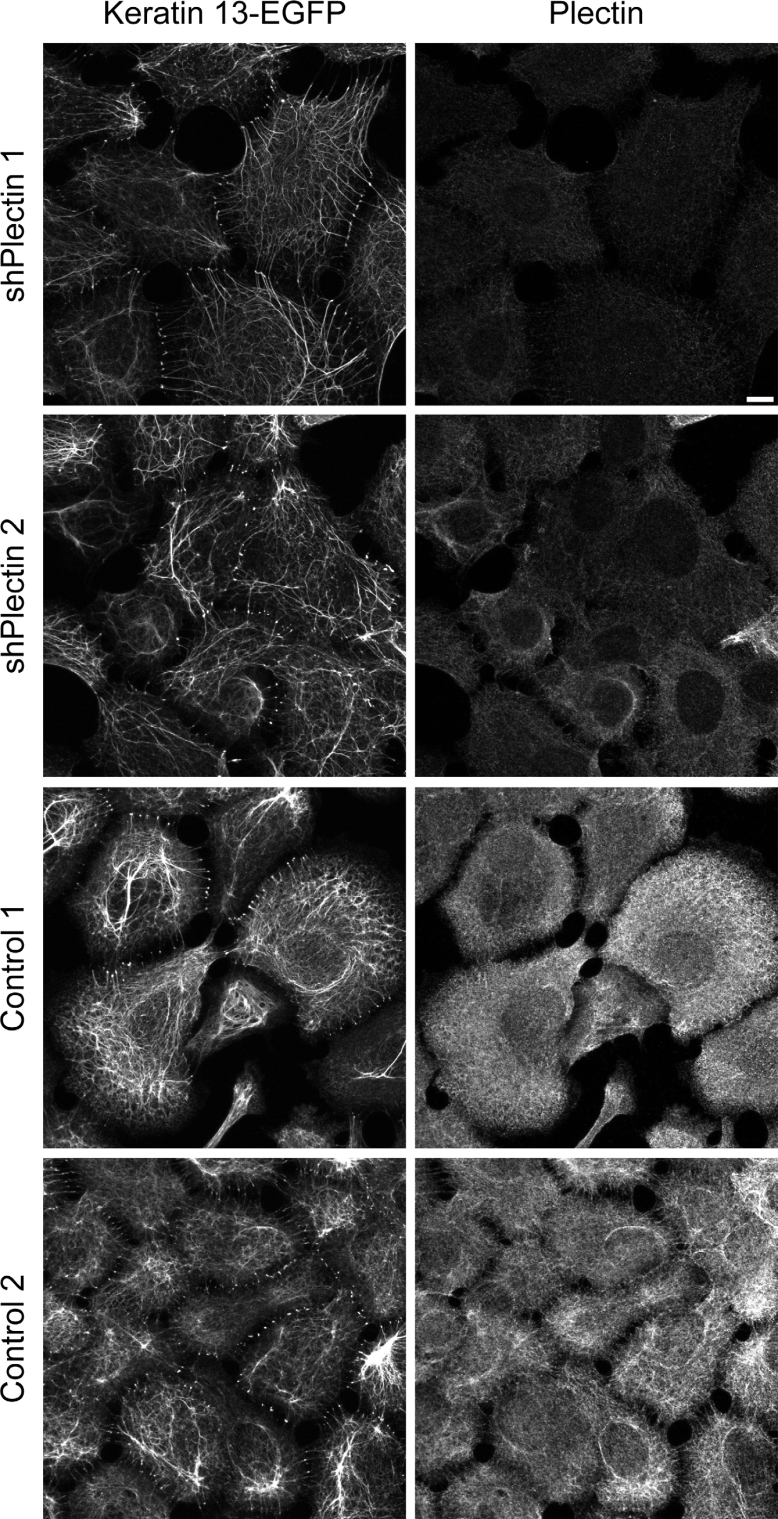
Immunofluorescence microscopy shows efficient down regulation of plectin in stably transfected A431 clones producing plectin shRNA. The fluorescence images (maximum intensity projections of complete cells) were recorded in AK13-1 subclones stably expressing HK13-EGFP and either scramble control shRNAs or plectin shRNAs. The cells were grown for 48 h on laminin 332-rich matrices in the absence of FCS prior to methanol/acetone fixation. Note the very low level of plectin immunoreactivity (guinea pig antibodies) in the clones expressing plectin shRNAs in contrast to the strong plectin signal in the scramble controls presenting filamentous fluorescence co-localizing in part with the endogenous keratin filaments. The HK13-EGFPnetwork is less dense and more compact in the plectin-deficient clones. Bar, 10 μm.

**Fig 4:**
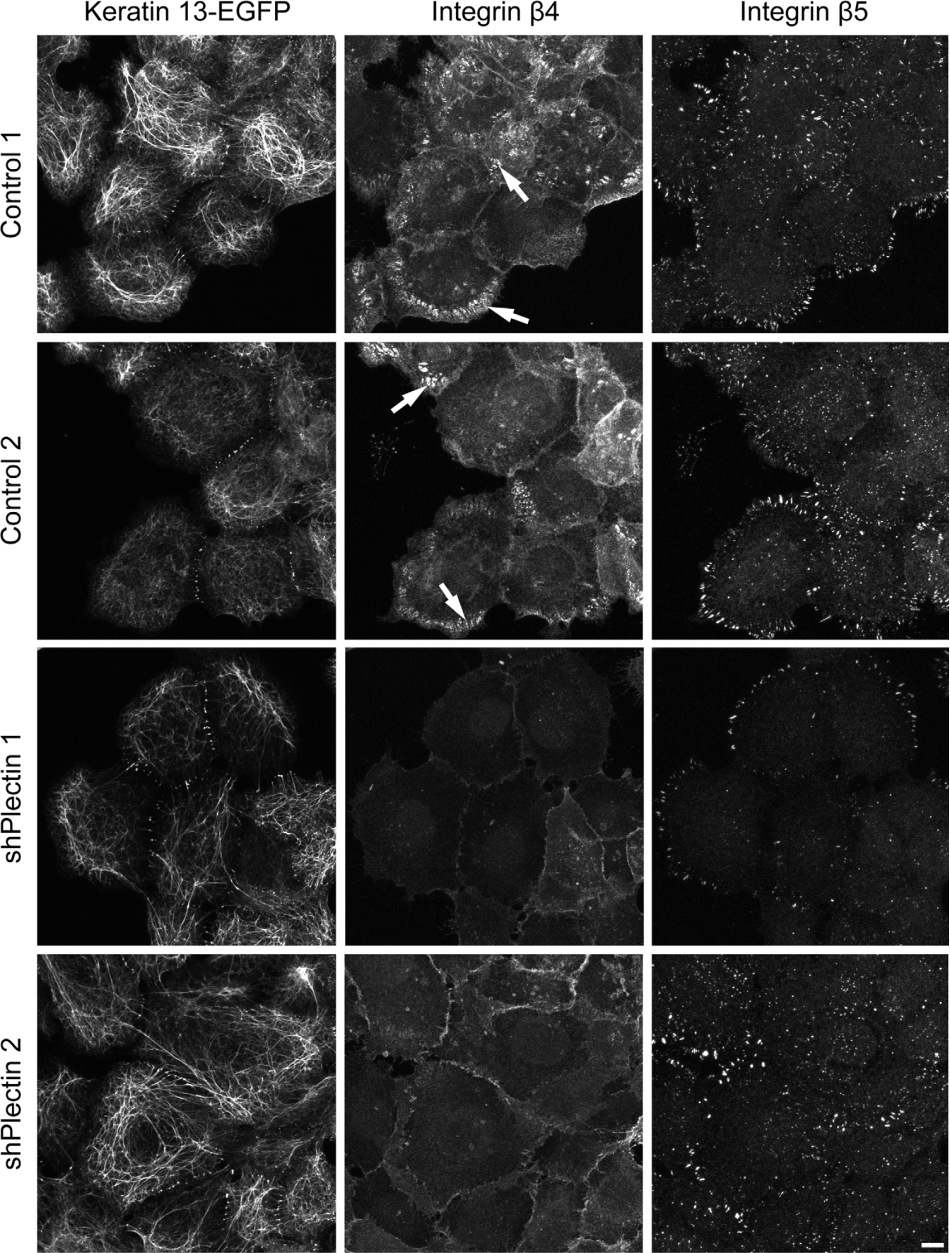
Integrin β4 clusters are almost absent in plectin-deficient cells while integrin β5 clusters persist. Immunocytochemistry of A431 clones stably expressing HK13-EGFP and either scramble control shRNA or plectin shRNA. Cells were cultivated for 48 h on laminin 332-rich matrix in the presence of FCS. The confocal images show only the bottom planes. The control cells present hemidesmosome-like integrin β4-positive clusters (arrows) whereas the plectin-deficient clones lack them and show only a rather weak and diffuse integrin β4 signal. Integrin β5 staining indicates the continued presence of focal adhesions in all clones. Keratin networks marked with HK13-EGFP are found to be barely perturbed in plectin-deficient clones. Bar, 10 μm.

Immunohistochemistry was performed next to examine keratin and actin network morphology. Again, keratin filament bundles appeared to be thicker and less dense than in the control and parent cell clones (Fig 3). Changes in actin stress fiber distribution were noticeable in large parts of the cell population, especially in cells that were connected to each other. Actin stress fibers were more abundant and longer than in control cells. They were also more often localized in the central cytosolic area (S3 Fig). Furthermore, integrin β4- and BPAG-1-positive hemidesmosomal structures were strongly reduced in plectin shRNA-producing clones 1 and 2 but not in the control cell clones (Fig 4 and S4 Fig). In plectin-depleted clones the integrin β4 signal was mostly restricted to cell borders. Other adhesion structures such as focal adhesions were present in all clones as shown by integrin β5 staining (Fig 4). Focal adhesions were also present in plectin-shRNA transfected HaCaT cells (S1B Fig).

### Keratin Filament Branch Length Is Increased upon Plectin Depletion Without Affecting Overall Cell Stiffness

**Fig 5:**
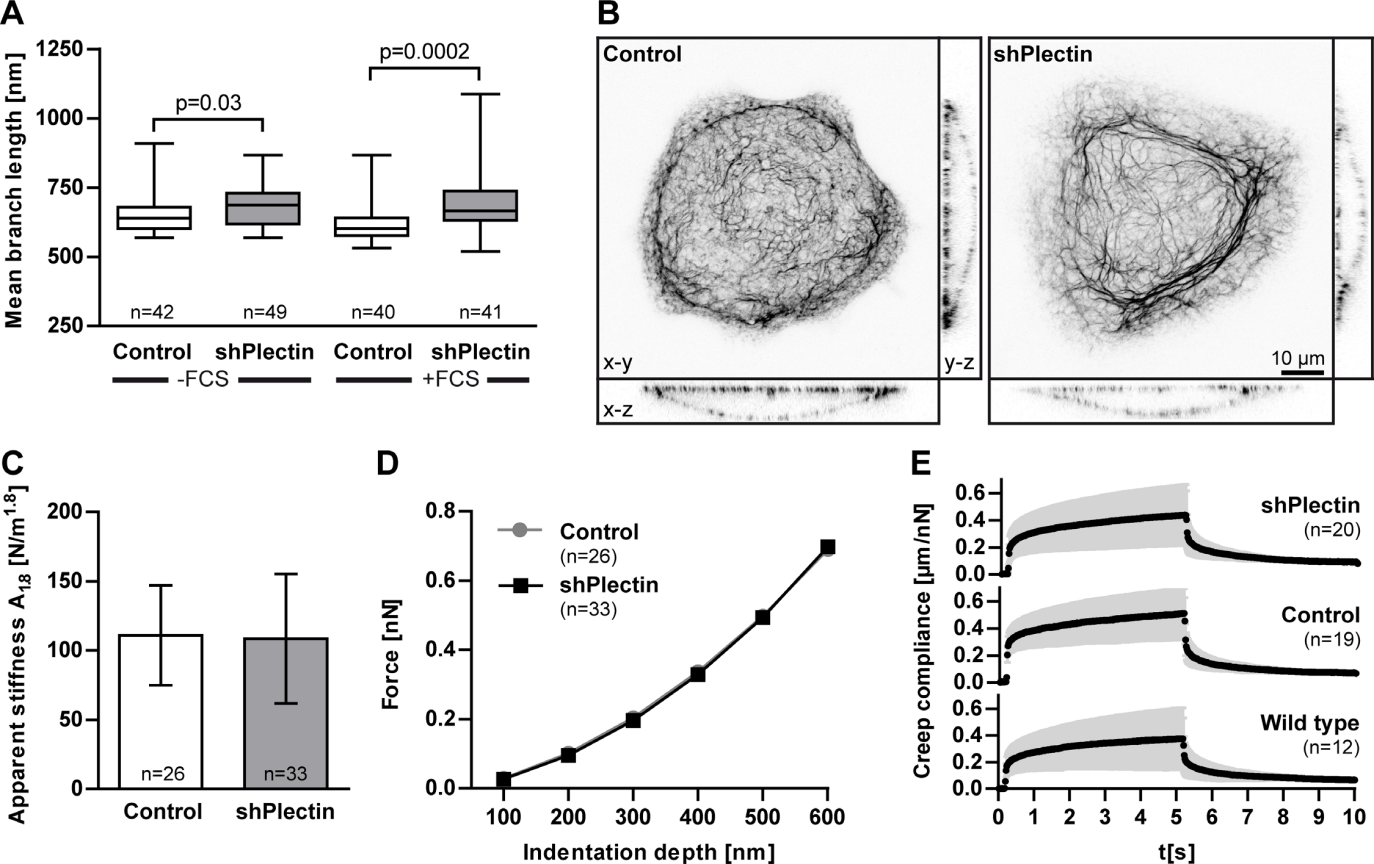
Plectin-deficiency results in decreased keratin network branch length but does not alter overall cellular stiffness and cytoplasmic viscoelasticity. A431 cells (wild type) and AK13-1 subclones stably expressing HK13-EGFP and either scramble control shRNAs or plectin shRNAs were grown for 48–57 h on laminin 332-rich matrices. **A** Histogram of branch length-measurements. Confocal fluorescence images of the bottom plane of isolated single cells were recorded to measure the HK13-EGFP filament length between branch points by automated image analysis. Results from either scramble control shRNA clones 1 and 2 or plectin shRNA clones 1 and 2 were combined. The plectin-deficient cells have significantly increased branch length in the absence of FCS which is even more apparent in the presence of FCS. The whiskers are min to max and statistical differences were determined by two-tailed Mann-Whitney test. **B** 3D confocal microscopy (maximum intensity projections are shown) of live cells after 48 h growth in the presence of 10% FCS. Note that mesh size and bundling of the keratin filament network is increased in the plectin-deficient cell. **C** Histogram depicting apparent stiffness A_1.8_ of single cells that was determined from indentation experiments that were performed above the nucleus in plectin shRNA clone 1 and scramble control clone 1. Error bars are standard deviation. **D** Graph showing the average forces needed to reach different indentation depths above the nucleus (same cells as in C). **E** Creep compliance measurements of magnetic tweezers experiments. The rescaled peaks [n_shPlectin_ = 117 (20 cells), n_control_ = 107 (19 cells), n_wild type_ = 67 (12 cells)] were fitted to equation 1 and averaged for each phenotype. Error bars are standard deviation. Note that no significant differences in viscoelastic properties of the cytoplasm were observed.

The response of keratin network morphology to plectin depletion was analyzed in more detail by computational analysis. To this end, keratin 13-EGFP fluorescence was recorded only in the bottom plane next to the coated glass surface in isolated well-spread single cells. The acquired fluorescence images from both plectin-depleted clones were superimposed and compared to superimposed images from scramble control clones. The comparison revealed that keratin branch length was significantly increased in plectin-depleted cells cultivated without FCS on a laminin 332-rich matrix for two days (Fig 5A). Branch length was also increased in the presence of FCS upon plectin downregulation. A comparison of the 3D keratin network organization of a control and a plectin-depleted cell is shown in Fig 5B.

To find out whether the altered branch length affects overall cellular stiffness, living cells were subjected to indentation experiments using atomic force microscopy. No differences were noted in plectin-depleted cells when the cantilever was applied near the nucleus (Fig 5C). In other experiments cytoplasmic viscoelasticity was tested by magnetic tweezers manipulation (Fig 5E). These experiments also did not reveal any significant differences upon plectin depletion, as indicated by the substantial overlap of averaged creep compliance curves and quantified by fitting equation 1 to the data. Fit parameters as averaged over all peaks for each cell type were J_0_ = 0.33±0.02 (wild type; error: standard error of the mean), 0.38±0.02 (shPlectin 1) and 0.39±0.02 (scramble shRNA control 1); β = 0.232±0.009 (wild type), 0.203±0.005 (shPlectin 1) and 0.185±0.005 (scramble shRNA control 1).

AFM measurements showed no difference for the resilience of control and plectin-depleted cells. The apparent stiffness of scramble shRNA control 1 cells (111±36 N/m²; error: standard deviation; n = 26) was nearly identical with the apparent stiffness of shPlectin 1 cells (108±47 N/m²; n = 33). The consistency of the resilience of the two cell types becomes even more obvious by plotting the applied force necessary to reach indentation depth of 100–600 nm (Fig 5D). For scrambled shRNA control 1 cells, the applied force to reach an indentation depth of 500 nm was 0.5±0.17 nN and 0.5±0.2 nN for shPlectin 1 cells.

### Plectin Is Not Needed for Keratin Cycling

**Fig 6:**
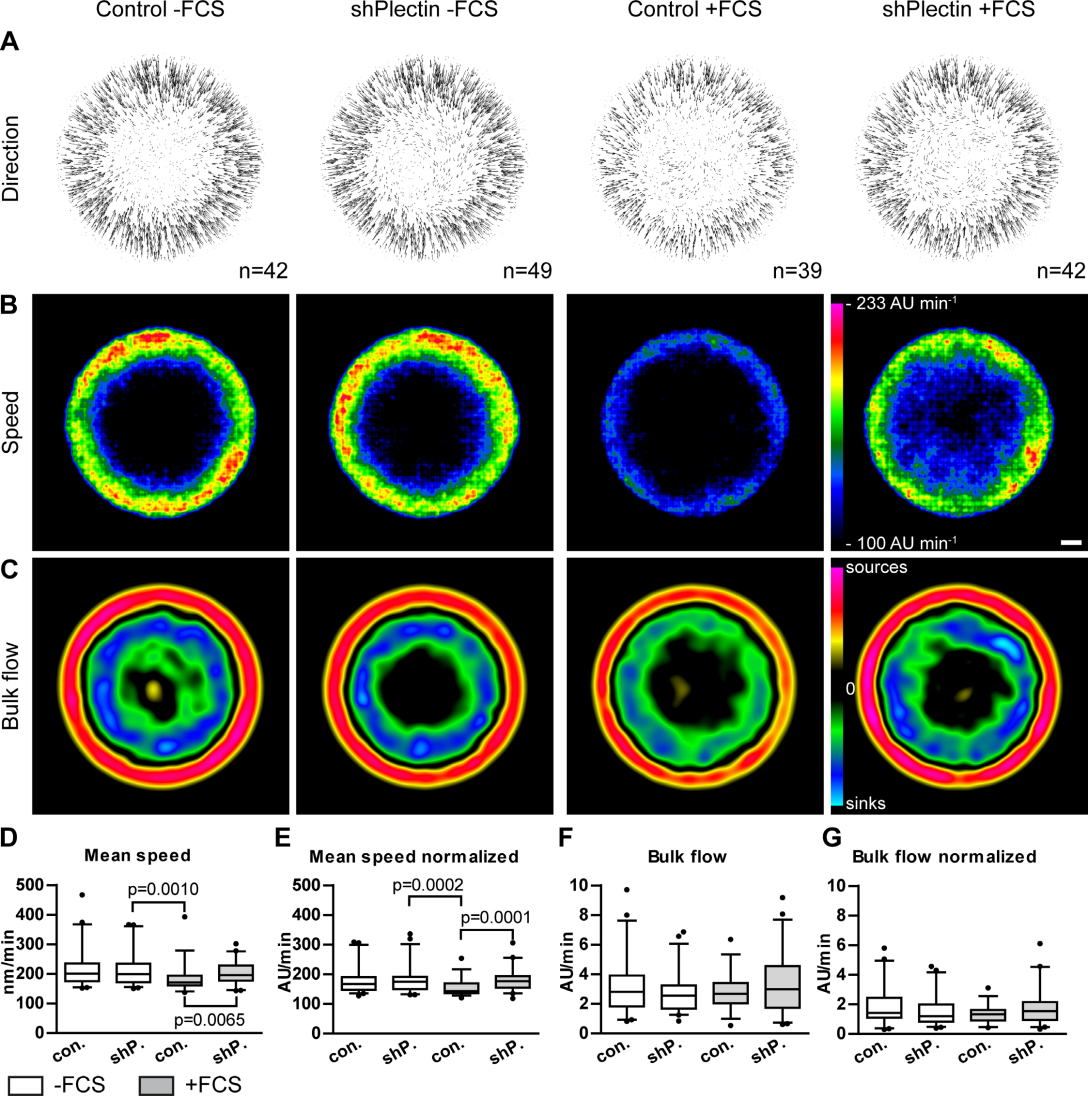
Keratin filament dynamics are affected by plectin-deficiency in the presence of serum but not in serum-starved cells. AK13-1 subclones stably expressing HK13-EGFP and either scramble control shRNAs (clones 1 and 2) or plectin shRNAs (clones 1 and 2) were cultivated for 48–57 h on laminin 332-rich matrices. HK13-EGFP dynamics were recorded by confocal microscopy in the bottom plane of isolated single cells at 30 s intervals for 10 minutes. For analysis, data from control clones and plectin shRNA clones were merged together in each case. **A-C** show averaged results from cells that were converted to standardized circular cells. The direction and speed of movement is represented by vector fields in the top panel. Color-coded heat maps of speed of keratin are depicted in the middle panel in arbitrary units (AU) per minute. The lower panel presents derived turnover maps revealing zones of keratin assembly (sources) and disassembly (sinks). Scale bar is 5000 AU. In **D-G** corresponding box plots for keratin mean speed and total bulk flow are shown as determined for non-normalized networks and normalized networks. The keratin movement in plectin-depleted cells is significantly increased in comparison to control cells cultivated in the presence of FCS, which induces the formation of hemidesmosome like structures. Otherwise, plectin has no influence on keratin movement in cells cultivated without FCS. Note that the detected differences in keratin turnover were too small to be significant. Statistical analysis was performed with two-tailed Mann-Whitney test and whiskers are 5–95%.

In the following experiments, keratin dynamics were compared in the presence and absence of plectin and in the presence and absence of FCS. Previously described image analysis tools and routines were used to calculate keratin motility and turnover from time-lapse recordings of keratin 13-EGFP fluorescence [9]. In comparison to the originally described method, slightly larger cells were recorded and recording parameters were optimized as described under methods. The data from both plectin-depleted clones were merged for comparison with merged data from both scramble control clones. Cellular fluorescence was recorded either under conditions that favor the formation of hemidesmosome-like structures, i.e., in the presence of FCS, or under conditions that prevent their formation, i.e., in the absence of FCS. To enable direct comparisons, keratin networks were normalized into a circular shape of identical diameter and were superimposed. The resulting vector fields of keratin movement (Fig 6A) show that keratin moved from the cell periphery toward the cell interior in each situation. The heat maps of keratin speed (Fig 6B) further revealed that keratin moves faster in the cell periphery and that almost no keratin movement is detectable in the cell center underneath the nucleus. These observations are fully compatible with previously reported data sets [9]. While the overall pattern was the same irrespective of the presence or absence of plectin and FCS, the slowest keratin motility was noted in control cells in the presence of FCS (Fig 6A and 6B). Of note, this is the only situation, in which hemidesmosome-like integrin β4-positive structures are present. The differences in mean speed are statistically significant using either non-normalized or normalized data sets (Fig 6D and 6E). Next, keratin 13-EGFP bulk flow was calculated by setting the keratin mass proportional to the fluorescence intensity of the moving filaments. Derived heat maps showed that keratin is mostly formed in the cell periphery and that it is disassembled primarily in a central area around the nucleus (Fig 6C). Between the assembly and disassembly zones no net keratin turnover was detected. This is also the case in the cell center underneath the nucleus where keratin filaments form the stable perinuclear cage. While a comparison of the heat maps suggests a slight increase in keratin turnover in cells lacking hemidesmosomal structures, this difference is not statistically significant when comparing total bulk flow (Fig 6F and 6G).

## Discussion

The keratin-rich epithelial A431 cell line was chosen for the experiments because it is a good model to study the relationship between plectin and keratin intermediate filaments. Transfected cell clones retained stable expression from multiple gene cassettes (keratin 13-EGFP, shRNA, ECFP). Lentiviral transduction was highly efficient. Advantageous for high resolution microscopy was that the cells are very flat (height ≈ 9 μm) on matrices such as laminin-332 (Fig 5B). The keratin network was mostly concentrated in the bottom plane of isolated fully spread A431 cells and also in cells within confluent monolayers. This focal plane is best accessible to confocal microscopy. The comparatively high degree of co-localization of plectin and keratin (Figs 1 and 3) was another advantage for the analysis of plectin's function for keratin network organization. The serum-responsiveness of hemidesmosomal structure formation offered a tool to differentiate between hemidesmosome-dependent from hemidesmosome-independent functions of plectin.

As expected, plectin downregulation led to altered keratin network organization. Our results are in accordance with previous observations of increased keratin filament bundle thickness and increased mesh size of the keratin network in keratinocytes lacking plectin [21]. The proposed function of plectin as an orthogonal cross-linker between keratin filaments is also in agreement with our finding that plectin depletion resulted in reduced keratin branching. Thus, plectin may be important for regulation of access to branch sites in keratin filaments and/or acts itself as an inducer of keratin branch formation. Examination of extracted keratin networks by immunostaining and scanning electron microscopy may help to shed more light on these properties [38]. It was surprising to find that the nuclear cage-like structure was still maintained in the absence of plectin, which has been suggested to anchor keratins to the nuclear envelope via nesprin 3a [20]. Otherwise, plectin-depleted clones formed longer actin stress fibers in central parts of the cell. This effect was especially noticeable in weakly contracted cell clusters (S3 Fig). A similar phenotype was described for plectin knockout keratinocytes [21] and keratin type II knockout keratinocytes that do not form keratin networks [39].

As expected, plectin deficiency induced loss of hemidesmosomal structures [16, 18]. Interestingly, changes in expression were not only observed for hemidesmosomal integrin β4 but also for integrins α3 and β1. Integrin β1 is known to pair with integrin α3 and also with integrins α2 and α5 all of which have been localized to focal adhesions [40]. The expression of the latter integrins, however, was not altered in plectin-depleted squamous cell carcinoma-derived A431 cells (Fig 2). Interestingly, α3β1 integrin and α6β4 integrin are both abundantly expressed in the basolateral membrane of basal keratinocytes in the epidermis [41] and serve as receptors for laminin 332 in the epidermis [42, 43]. A cross talk has been proposed for hemidesmosomal integrins α6β4 and focal contact-localized integrins α3β1 in epidermal keratinocytes and squamous cell carcinomas involving CD151 and plectin with complex consequences for cell migration [44, 45]. Furthermore, α3/β1 integrin depletion has been shown to increase migration and adhesion of keratinocytes [46, 47]. In accordance, plectin-deficiency has been shown to increase keratinocyte migration [21]. Thus, it has been suggested that reduced plectin levels are also responsible for the observed increased migration of keratin-free keratinocytes [48]. However, we did not detect changes in cell mechanics in living A431 cells upon plectin depletion (for similar results see [49]). Probing cells from the outside by AFM did not reveal differences in cell rigidity at the position of the nucleus and probing cytoplasmic viscoelasticity from within the cell using magnetic tweezers did also not reveal differences in cellular biomechanics. We cannot exclude, however, that more sensitive tools will detect differences especially in the basal cell compartment which contains the majority of the keratin cytoskeleton.

The main finding of the current work is that plectin is not a major determinant of keratin cycling:

- Keratins continue to be nucleated, which has been shown to occur in close vicinity to focal adhesions [50]. In the case of vimentin, a direct relationship had been demonstrated for focal adhesion-localized plectin 1f and filament formation [23]. Whether the differences are due of intermediate filament type-specific properties or are a consequence of cell type-specific expression of plectin splice variants [51] remains to be shown.
- Actin-dependent inward movement of newly-formed keratin particles continues. It had been suggested that actin retrograde flow may be coupled to inward movement through plectin and that similar mechanisms may be relevant for dynamic microtubule-keratin interactions [6].
- Bundling of keratins is obviously affected by plectin but does not influence keratin turnover measurably. This suggests that bundle formation is not sufficient to protect keratins from disassembly. Similarly, the large keratin granules observed in cells producing mutant keratins have a much higher turnover rate than typical keratin filaments [7, 52].
- Turnover of keratin filaments is not measurably affected by plectin depletion, although keratin motility is increased. A possible explanation for the apparent discrepancy is that loss of plectin does not result in global alterations of keratin cycling but affects only a subset of keratins, i.e. those that are released by disassembly of hemidesmosome-like structures. Their entry into the turnover cycle may not suffice to be detected quantitatively by our measuring methods. Turnover analyses at the single filament level will help to clarify this issue.

Possible reasons for the comparatively mild effects of plectin depletion on keratin cycling and network organization are differences between transformed and non-transformed cells and molecular redundancy. Multiple plakins are expressed in epithelial tissues, some of which have been implicated in keratin network organization [53–55]. Furthermore, other mechanisms may contribute to keratin dynamics such as disulfide bounding which has been shown to stabilize K5/K14 networks [11].

## Supporting Information

S1 Fig Transient downregulation of plectin by shRNA in HaCaT keratinocytes. HaCaT cells were infected two weeks prior to experiments with shRNAs targeting all plectin isoforms. The cells were grown for 72 hours on uncoated glass surfaces and were methanol/acetone fixed for analysis by immunocytochemistry. Cells that were transfected with shRNAs are marked with asterisks and show nearly absent plectin signal in comparison to neighboring cells that were not transfected. **A** Integrin β4-positive hemidesmosome-like structures are marked with arrows in cells where plectin was not downregulated. These structures are absent in transfected cells (see also B). The keratin network was stained with pan-keratin antibodies and shows similar overall organization in cells with or without plectin. **B** Integrin β5-positive focal adhesions are localized between hemidesmosome-like structures. In plectin knock down cells the latter are absent but the localization of focal adhesions appears to be altered. Plectin was detected with guinea pig antibodies. Bars, 10 μm.
(PDF)

S2 Fig Plectin downregulation does not affect keratin isoform levels. The lysates of plectin knock down AK13-1 clones tested in this immunoblot are the same as in Fig 1 [scramble shRNA clones 1 and 2 (controls), plectin shRNA clones 1 and 2 (shPlectin), and wild type A431 cells (A431)]. The detected alternations in keratin protein levels are not related to plectin downregulation as they are not consistent in all clones.
(PDF)

S3 Fig Altered actin stress fiber localization upon plectin downregulation. The fluorescence images (maximum intensity projections of complete cells) were recorded in AK13-1 subclones stably expressing either scramble control shRNAs or plectin shRNAs. The cells were grown for 48 h on laminin 332-rich matrices in the presence of FCS prior to paraformaldehyde fixation. Filamentous actin was stained with phalloidin. **A** shows that isolated plectin-depleted cells form slightly longer actin stress fibers than control cells (arrows). **B** shows images of cell clusters. Note the increase in cytosolic actin stress fibers in the plectin-deficient cells. **C** depicts examples of extreme cytosolic actin stress fiber localization in shPlectin clone 1. Bars, 10 μm.
(PDF)

S4 Fig Formation of BPAG-1- and integrin β4- positive hemidesmosomal structures is strongly decreased upon plectin downregulation. The fluorescence images (maximum intensity projections of basal cell compartment) were recorded in scramble control shRNA clone 1 (top) and plectin shRNA clone 1 (bottom). Cells were grown for 48 h on laminin 332-rich matrices in the presence of FCS prior to methanol/acetone fixation. Note that the colocalized BPAG-1- and integrin β4- staining is strongly reduced in plectin shRNA clone 1. Plectin was detected with guinea pig antibodies. Bar, 10 μm.
(PDF)

S1 Files Uncropped immunoblot recordings without contrast adjustment, measurements used for diagrams, and secondary antibodies used. Exposures of immunoblot membranes 1, 2, and 3 were used for Fig 2 and exposures of membranes 3, 4 and 5 for S2 Fig. The immunoblot TIFF files are ordered according to stripping steps (1 = before stripping). The positions of the co-electrophoresed size markers were inserted with FusionCapt Advance software version 16.06 on a Fusion-Solo.WL.4M (Vilber Lourmat). The exact details on the ProSieve QuadColor Protein Marker 4.6–300 kDa can be found on the manufacturer’s homepage at http://www.lonza.com/products-services/bio-research/electrophoresis-of-nucleic-acids-and-proteins/protein-electrophoresis/protein-stains-markers/prosieve-protein-colored-and-unstained-markers.aspx. The polypeptides remaining in the SDS-polyacrylamide gels after blotting onto the PVDF membranes were detected with a colloidal staining solution [20 mM CuSO4, 10% (v/v) acetic acid, 45% (v/v) methanol, 0.15% (w/v) Coomassie Brilliant Blue G250 (SERVA Electrophoresis)] and unbound dye was removed by washing in water. Stained proteins were recorded on a Quantum ST4 1100/26MX (Vilber Lourmat) using Quantum-Capt software version 15.12 to estimate transfer efficiency. They are also included as TIFF files. Measurements used for diagrams and statistical analyses in Fig 5A, 5C, 5D, 5E and Fig 6D are deposited in the measurements.xlsx file. Detailed information about secondary antibodies is included in antibodies.pdf.
(ZIP)
