# Supplementary figures and images for: Effects of Plectin Depletion on Keratin Network Dynamics and Organization
Source: PLoS One. 2016 Mar 23;11(3):e0149106. doi: 10.1371/journal.pone.0149106 (PMC4805305; doi:10.1371/journal.pone.0149106)

**A**

Plectin

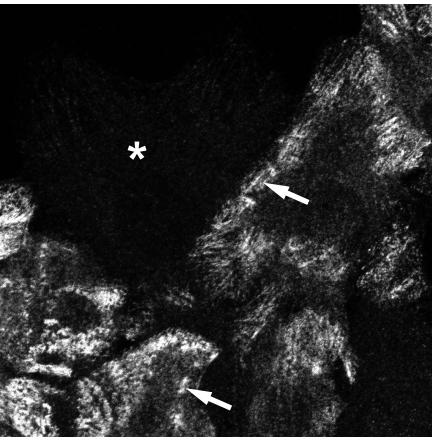Integrin  $\beta 4$ 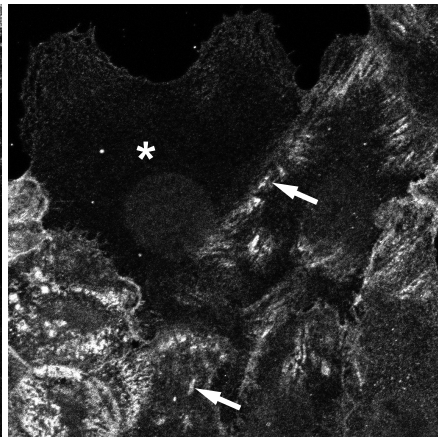

Keratin

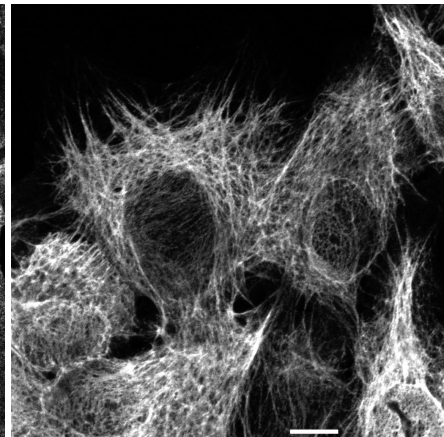**B**

Plectin

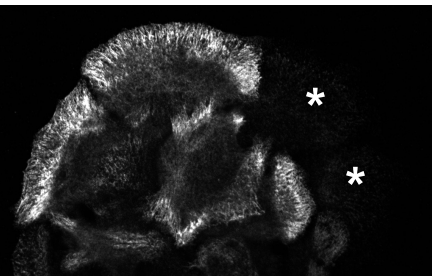Integrin  $\beta 4$ 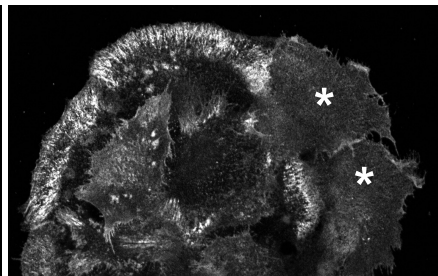Integrin  $\beta 5$ 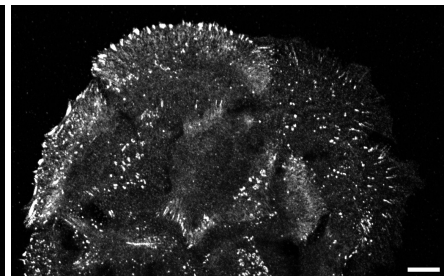

Supplement: S1 Fig — HaCaT cells were infected two weeks prior to experiments with shRNAs targeting all plectin isoforms. The cells were grown for 72 hours on uncoated glass surfaces and were methanol/acetone fixed for analysis by immunocytochemistry. Cells that were transfected with shRNAs are marked with asterisks and show nearly absent plectin signal in comparison to neighboring cells that were not transfected. A Integrin β4-positive hemidesmosome-like structures are marked with arrows in cells where plectin was not downregulated. These structures are absent in transfected cells (see also B). The keratin network was stained with pan-keratin antibodies and shows similar overall organization in cells with or without plectin. B Integrin β5-positive focal adhesions are localized between hemidesmosome-like structures. In plectin knock down cells the latter are absent but the localization of focal adhesions appears to be altered. Plectin was detected with guinea pig antibodies. Bars, 10 μm. (PDF) [file pone.0149106.s001.pdf]

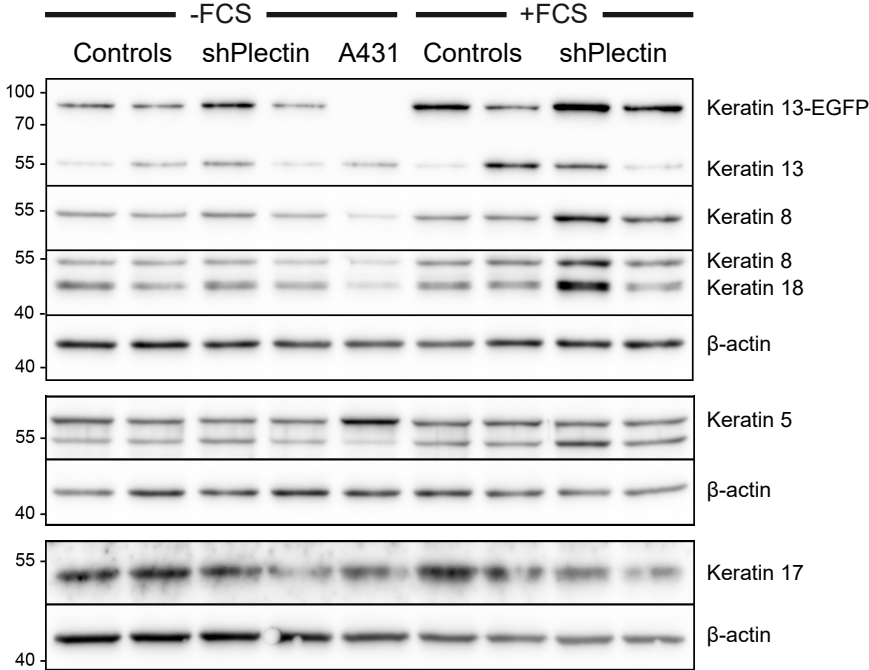

Supplement: S2 Fig — The lysates of plectin knock down AK13-1 clones tested in this immunoblot are the same as in Fig 1 [scramble shRNA clones 1 and 2 (controls), plectin shRNA clones 1 and 2 (shPlectin), and wild type A431 cells (A431)]. The detected alternations in keratin protein levels are not related to plectin downregulation as they are not consistent in all clones. (PDF) [file pone.0149106.s002.pdf]

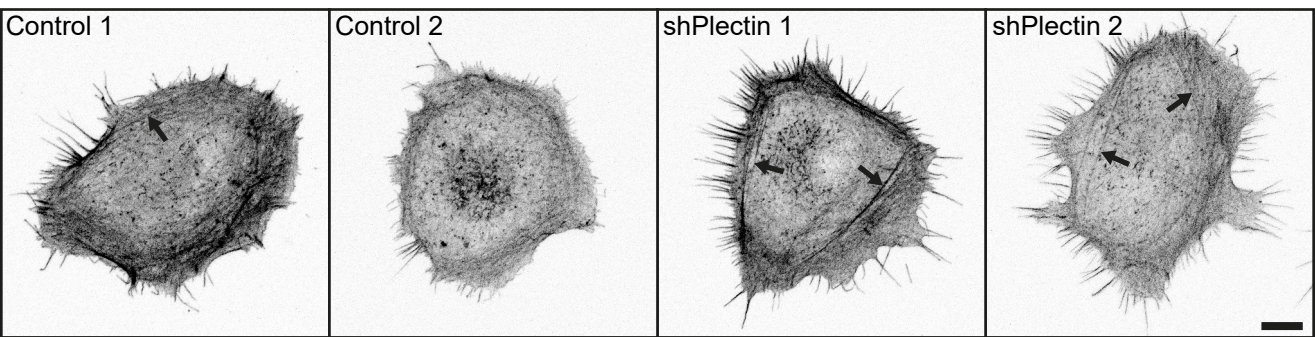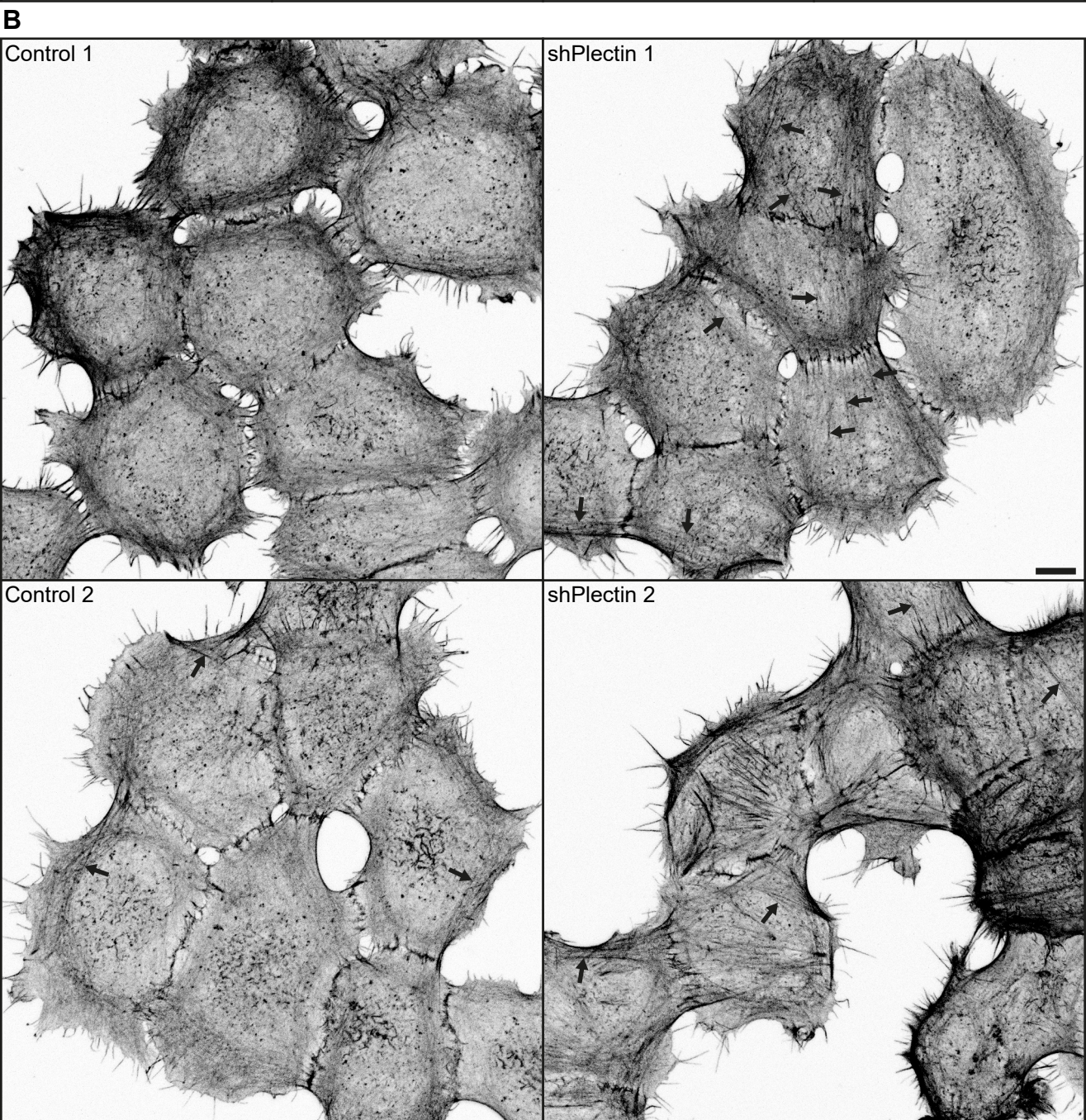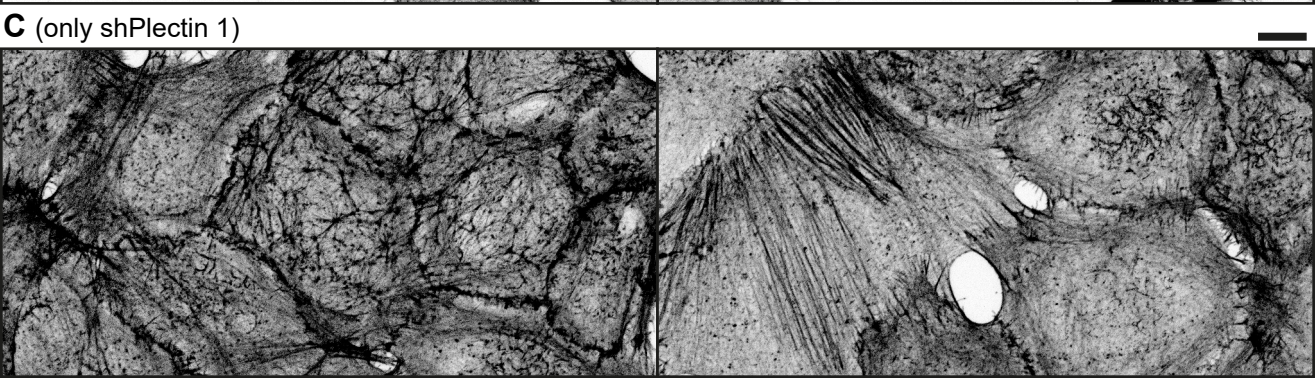

Supplement: S3 Fig — The fluorescence images (maximum intensity projections of complete cells) were recorded in AK13-1 subclones stably expressing either scramble control shRNAs or plectin shRNAs. The cells were grown for 48 h on laminin 332-rich matrices in the presence of FCS prior to paraformaldehyde fixation. Filamentous actin was stained with phalloidin. A shows that isolated plectin-depleted cells form slightly longer actin stress fibers than control cells (arrows). B shows images of cell clusters. Note the increase in cytosolic actin stress fibers in the plectin-deficient cells. C depicts examples of extreme cytosolic actin stress fiber localization in shPlectin clone 1. Bars, 10 μm. (PDF) [file pone.0149106.s003.pdf]

BPAG-1

Integrin  $\beta 4$

Plectin

Keratin 13-EGFP

Control

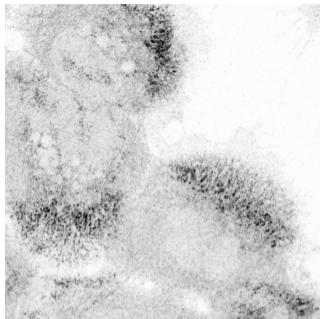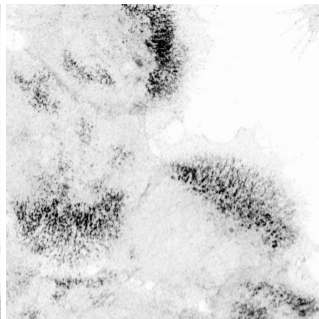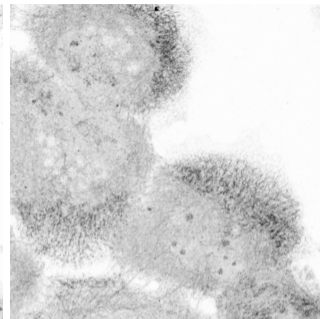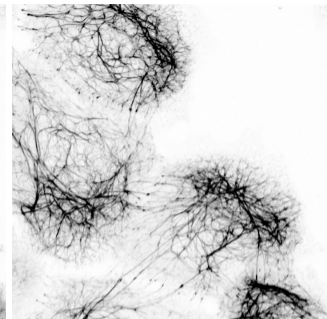

shPlectin

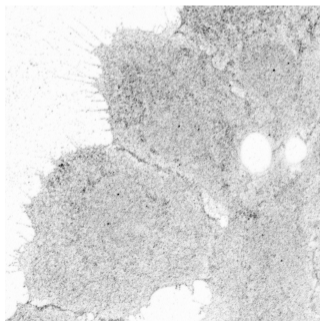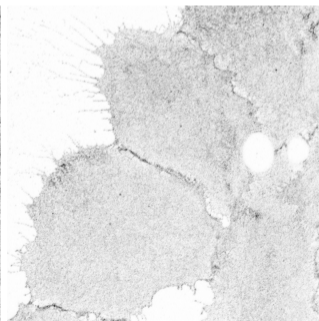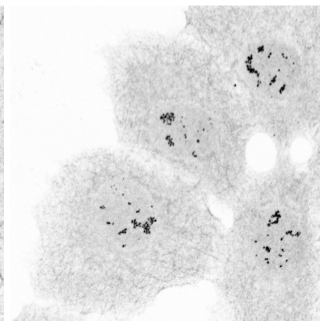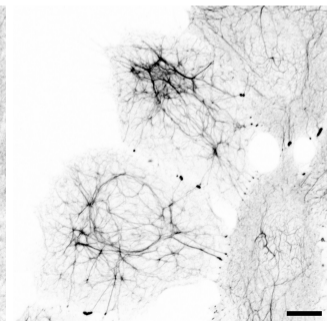

Supplement: S4 Fig — The fluorescence images (maximum intensity projections of basal cell compartment) were recorded in scramble control shRNA clone 1 (top) and plectin shRNA clone 1 (bottom). Cells were grown for 48 h on laminin 332-rich matrices in the presence of FCS prior to methanol/acetone fixation. Note that the colocalized BPAG-1- and integrin β4- staining is strongly reduced in plectin shRNA clone 1. Plectin was detected with guinea pig antibodies. Bar, 10 μm. (PDF) [file pone.0149106.s004.pdf]

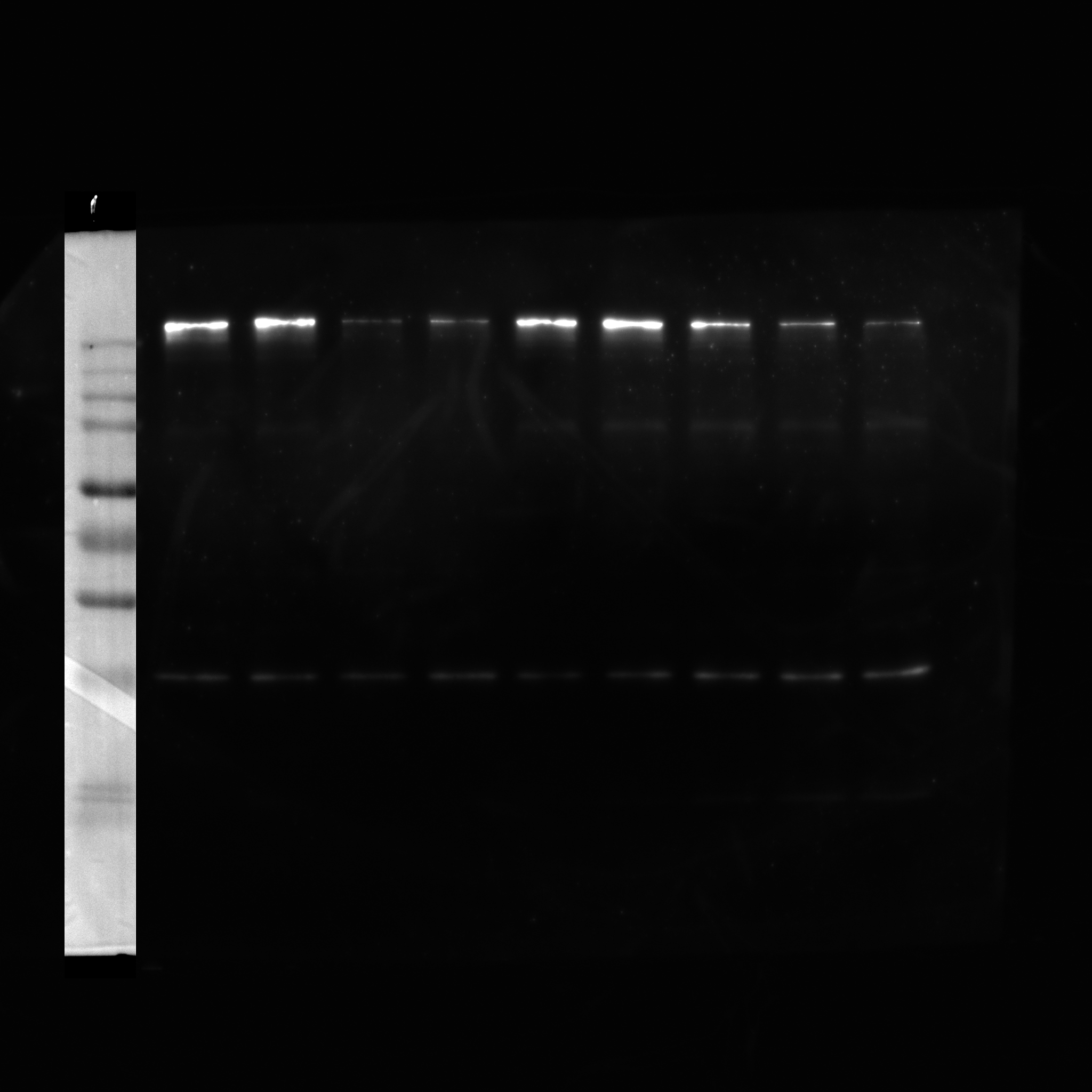

Supplement: S1 Files — Exposures of immunoblot membranes 1, 2, and 3 were used for Fig 2 and exposures of membranes 3, 4 and 5 for S2 Fig. The immunoblot TIFF files are ordered according to stripping steps (1 = before stripping). The positions of the co-electrophoresed size markers were inserted with FusionCapt Advance software version 16.06 on a Fusion-Solo.WL.4M (Vilber Lourmat). The exact details on the ProSieve QuadColor Protein Marker 4.6–300 kDa can be found on the manufacturer’s homepage at http://www.lonza.com/products-services/bio-research/electrophoresis-of-nucleic-acids-and-proteins/protein-electrophoresis/protein-stains-markers/prosieve-protein-colored-and-unstained-markers.aspx. The polypeptides remaining in the SDS-polyacrylamide gels after blotting onto the PVDF membranes were detected with a colloidal staining solution [20 mM CuSO4, 10% (v/v) acetic acid, 45% (v/v) methanol, 0.15% (w/v) Coomassie Brilliant Blue G250 (SERVA Electrophoresis)] and unbound dye was removed by washing in water. Stained proteins were recorded on a Quantum ST4 1100/26MX (Vilber Lourmat) using Quantum-Capt software version 15.12 to estimate transfer efficiency. They are also included as TIFF files. Measurements used for diagrams and statistical analyses in Fig 5A, 5C, 5D, 5E and Fig 6D are deposited in the measurements.xlsx file. Detailed information about secondary antibodies is included in antibodies.pdf. (ZIP) [file pone.0149106.s005.zip › Membrane 1/140213_1_plectin HD1_+ruler.Tif]

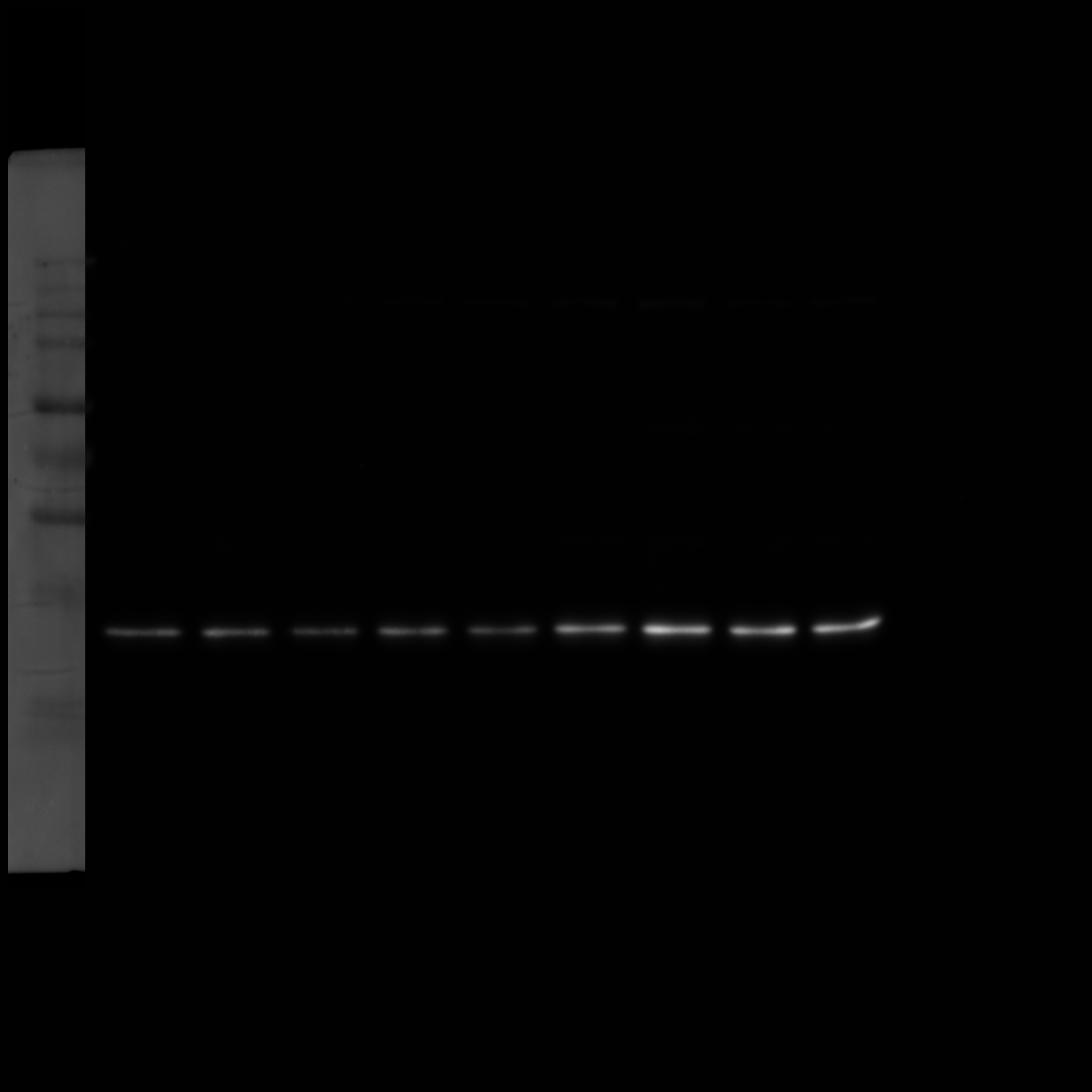

Supplement: S1 Files — Exposures of immunoblot membranes 1, 2, and 3 were used for Fig 2 and exposures of membranes 3, 4 and 5 for S2 Fig. The immunoblot TIFF files are ordered according to stripping steps (1 = before stripping). The positions of the co-electrophoresed size markers were inserted with FusionCapt Advance software version 16.06 on a Fusion-Solo.WL.4M (Vilber Lourmat). The exact details on the ProSieve QuadColor Protein Marker 4.6–300 kDa can be found on the manufacturer’s homepage at http://www.lonza.com/products-services/bio-research/electrophoresis-of-nucleic-acids-and-proteins/protein-electrophoresis/protein-stains-markers/prosieve-protein-colored-and-unstained-markers.aspx. The polypeptides remaining in the SDS-polyacrylamide gels after blotting onto the PVDF membranes were detected with a colloidal staining solution [20 mM CuSO4, 10% (v/v) acetic acid, 45% (v/v) methanol, 0.15% (w/v) Coomassie Brilliant Blue G250 (SERVA Electrophoresis)] and unbound dye was removed by washing in water. Stained proteins were recorded on a Quantum ST4 1100/26MX (Vilber Lourmat) using Quantum-Capt software version 15.12 to estimate transfer efficiency. They are also included as TIFF files. Measurements used for diagrams and statistical analyses in Fig 5A, 5C, 5D, 5E and Fig 6D are deposited in the measurements.xlsx file. Detailed information about secondary antibodies is included in antibodies.pdf. (ZIP) [file pone.0149106.s005.zip › Membrane 1/140213_2_GAPDH_+ruler.Tif]

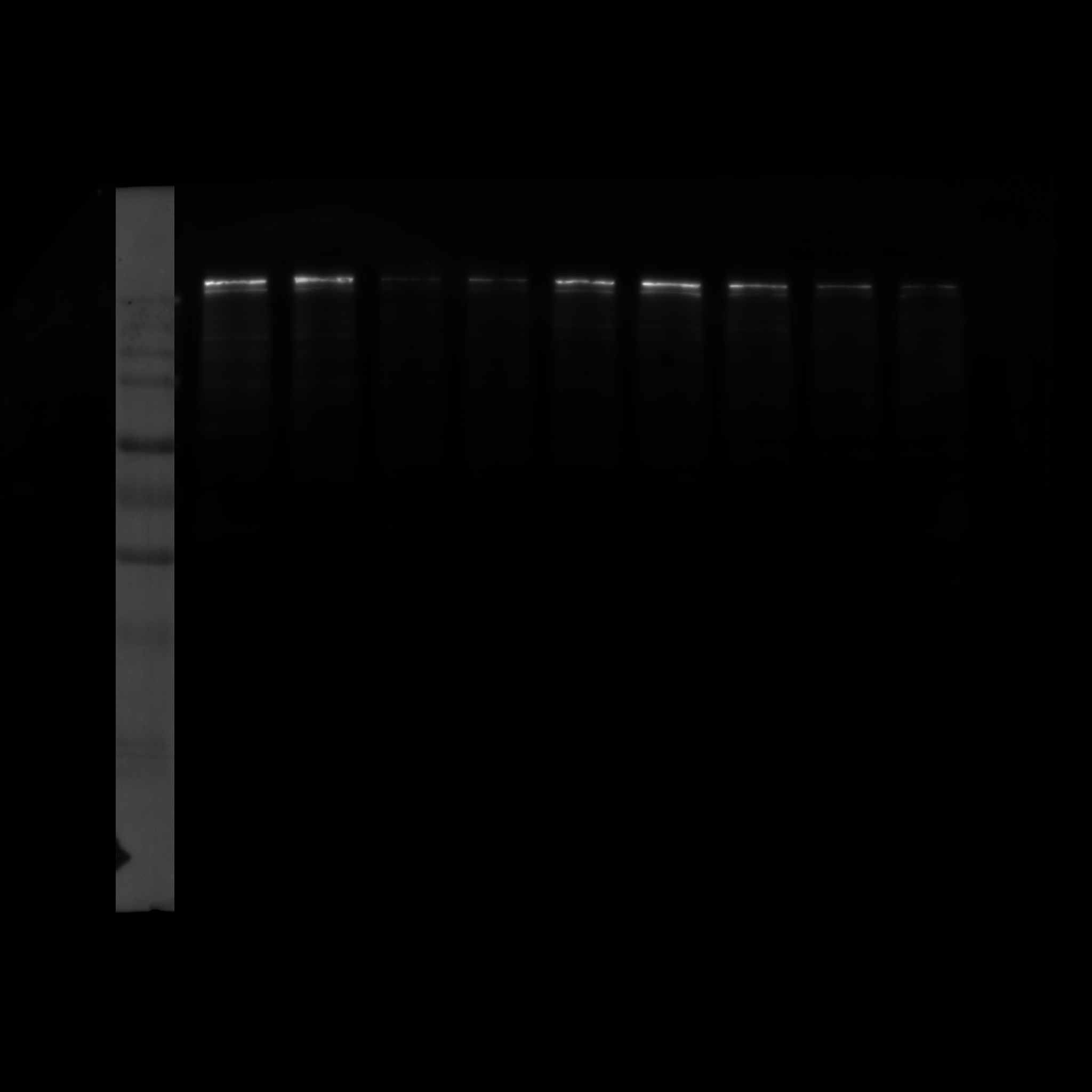

Supplement: S1 Files — Exposures of immunoblot membranes 1, 2, and 3 were used for Fig 2 and exposures of membranes 3, 4 and 5 for S2 Fig. The immunoblot TIFF files are ordered according to stripping steps (1 = before stripping). The positions of the co-electrophoresed size markers were inserted with FusionCapt Advance software version 16.06 on a Fusion-Solo.WL.4M (Vilber Lourmat). The exact details on the ProSieve QuadColor Protein Marker 4.6–300 kDa can be found on the manufacturer’s homepage at http://www.lonza.com/products-services/bio-research/electrophoresis-of-nucleic-acids-and-proteins/protein-electrophoresis/protein-stains-markers/prosieve-protein-colored-and-unstained-markers.aspx. The polypeptides remaining in the SDS-polyacrylamide gels after blotting onto the PVDF membranes were detected with a colloidal staining solution [20 mM CuSO4, 10% (v/v) acetic acid, 45% (v/v) methanol, 0.15% (w/v) Coomassie Brilliant Blue G250 (SERVA Electrophoresis)] and unbound dye was removed by washing in water. Stained proteins were recorded on a Quantum ST4 1100/26MX (Vilber Lourmat) using Quantum-Capt software version 15.12 to estimate transfer efficiency. They are also included as TIFF files. Measurements used for diagrams and statistical analyses in Fig 5A, 5C, 5D, 5E and Fig 6D are deposited in the measurements.xlsx file. Detailed information about secondary antibodies is included in antibodies.pdf. (ZIP) [file pone.0149106.s005.zip › Membrane 1/140213_3_plectin_+ruler.Tif]

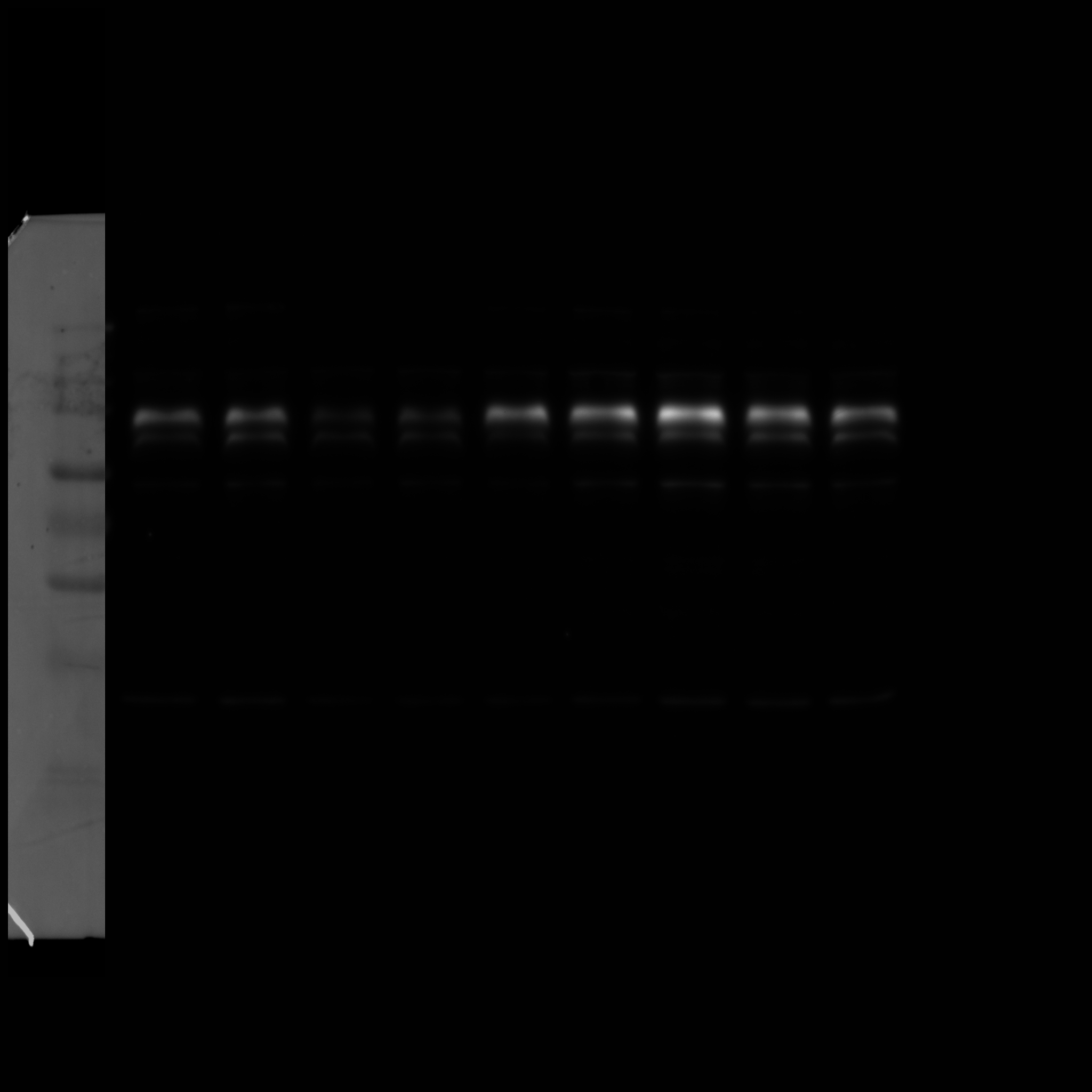

Supplement: S1 Files — Exposures of immunoblot membranes 1, 2, and 3 were used for Fig 2 and exposures of membranes 3, 4 and 5 for S2 Fig. The immunoblot TIFF files are ordered according to stripping steps (1 = before stripping). The positions of the co-electrophoresed size markers were inserted with FusionCapt Advance software version 16.06 on a Fusion-Solo.WL.4M (Vilber Lourmat). The exact details on the ProSieve QuadColor Protein Marker 4.6–300 kDa can be found on the manufacturer’s homepage at http://www.lonza.com/products-services/bio-research/electrophoresis-of-nucleic-acids-and-proteins/protein-electrophoresis/protein-stains-markers/prosieve-protein-colored-and-unstained-markers.aspx. The polypeptides remaining in the SDS-polyacrylamide gels after blotting onto the PVDF membranes were detected with a colloidal staining solution [20 mM CuSO4, 10% (v/v) acetic acid, 45% (v/v) methanol, 0.15% (w/v) Coomassie Brilliant Blue G250 (SERVA Electrophoresis)] and unbound dye was removed by washing in water. Stained proteins were recorded on a Quantum ST4 1100/26MX (Vilber Lourmat) using Quantum-Capt software version 15.12 to estimate transfer efficiency. They are also included as TIFF files. Measurements used for diagrams and statistical analyses in Fig 5A, 5C, 5D, 5E and Fig 6D are deposited in the measurements.xlsx file. Detailed information about secondary antibodies is included in antibodies.pdf. (ZIP) [file pone.0149106.s005.zip › Membrane 1/140213_4_integrin b1_+ruler.Tif]

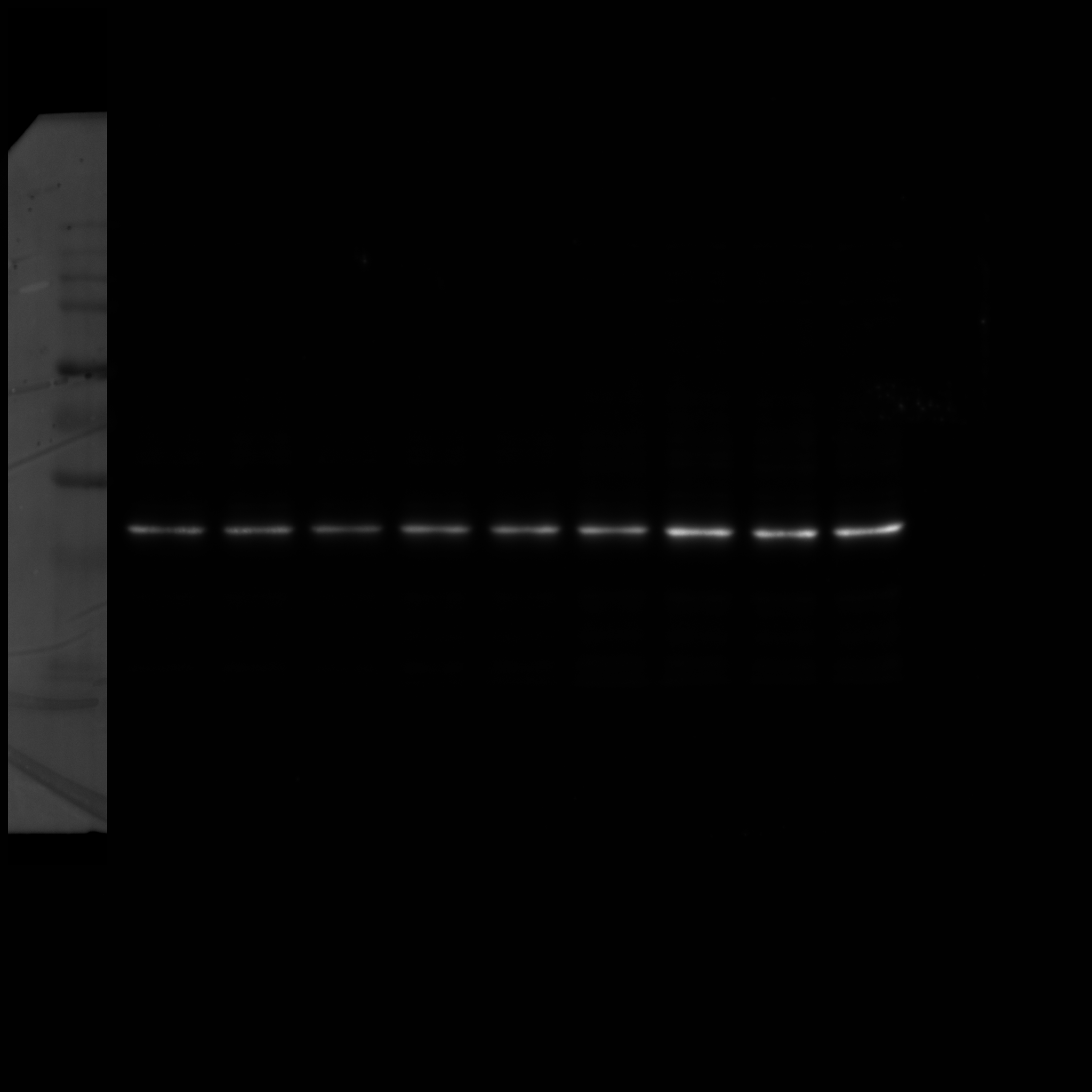

Supplement: S1 Files — Exposures of immunoblot membranes 1, 2, and 3 were used for Fig 2 and exposures of membranes 3, 4 and 5 for S2 Fig. The immunoblot TIFF files are ordered according to stripping steps (1 = before stripping). The positions of the co-electrophoresed size markers were inserted with FusionCapt Advance software version 16.06 on a Fusion-Solo.WL.4M (Vilber Lourmat). The exact details on the ProSieve QuadColor Protein Marker 4.6–300 kDa can be found on the manufacturer’s homepage at http://www.lonza.com/products-services/bio-research/electrophoresis-of-nucleic-acids-and-proteins/protein-electrophoresis/protein-stains-markers/prosieve-protein-colored-and-unstained-markers.aspx. The polypeptides remaining in the SDS-polyacrylamide gels after blotting onto the PVDF membranes were detected with a colloidal staining solution [20 mM CuSO4, 10% (v/v) acetic acid, 45% (v/v) methanol, 0.15% (w/v) Coomassie Brilliant Blue G250 (SERVA Electrophoresis)] and unbound dye was removed by washing in water. Stained proteins were recorded on a Quantum ST4 1100/26MX (Vilber Lourmat) using Quantum-Capt software version 15.12 to estimate transfer efficiency. They are also included as TIFF files. Measurements used for diagrams and statistical analyses in Fig 5A, 5C, 5D, 5E and Fig 6D are deposited in the measurements.xlsx file. Detailed information about secondary antibodies is included in antibodies.pdf. (ZIP) [file pone.0149106.s005.zip › Membrane 1/140213_5_actin_+ruler.Tif]

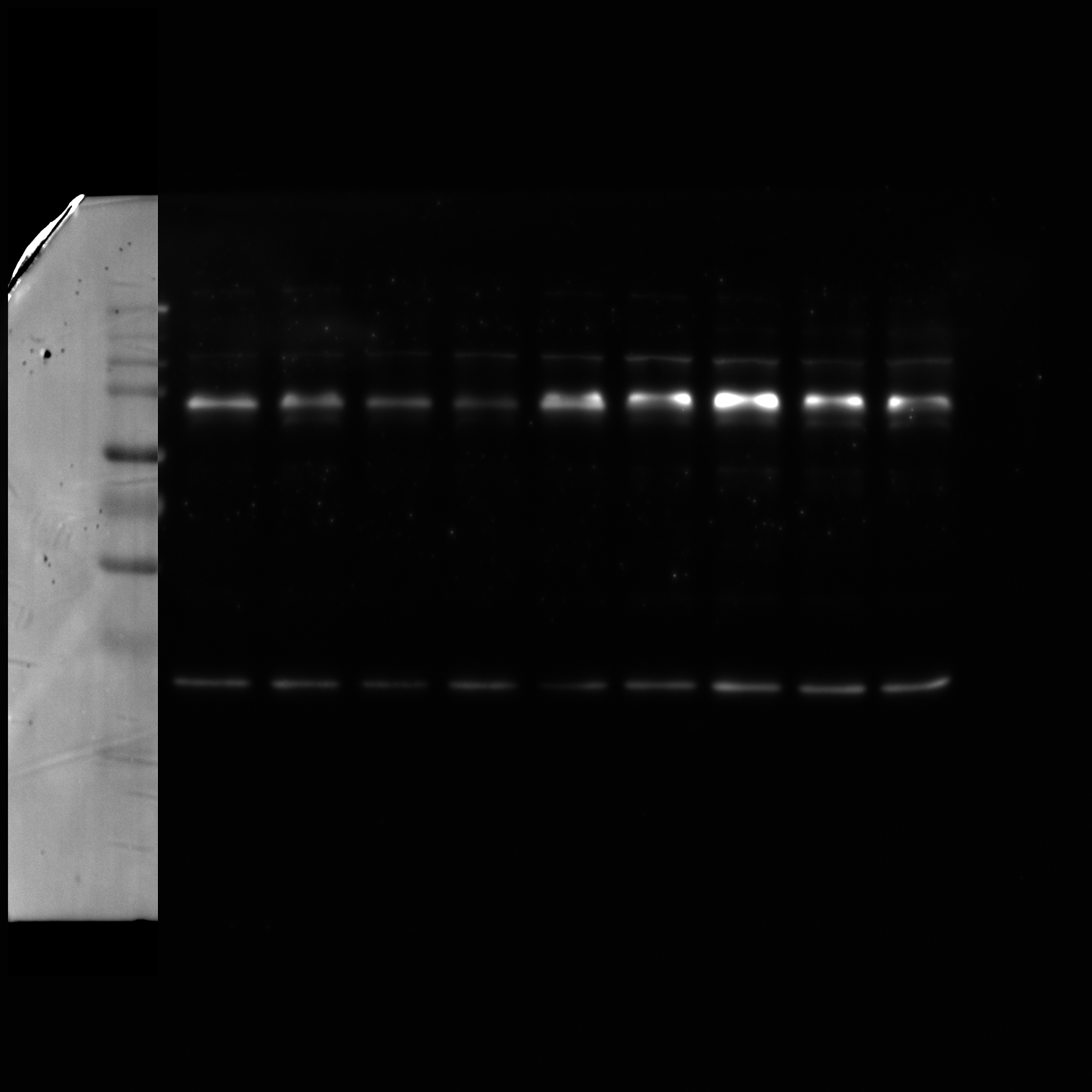

Supplement: S1 Files — Exposures of immunoblot membranes 1, 2, and 3 were used for Fig 2 and exposures of membranes 3, 4 and 5 for S2 Fig. The immunoblot TIFF files are ordered according to stripping steps (1 = before stripping). The positions of the co-electrophoresed size markers were inserted with FusionCapt Advance software version 16.06 on a Fusion-Solo.WL.4M (Vilber Lourmat). The exact details on the ProSieve QuadColor Protein Marker 4.6–300 kDa can be found on the manufacturer’s homepage at http://www.lonza.com/products-services/bio-research/electrophoresis-of-nucleic-acids-and-proteins/protein-electrophoresis/protein-stains-markers/prosieve-protein-colored-and-unstained-markers.aspx. The polypeptides remaining in the SDS-polyacrylamide gels after blotting onto the PVDF membranes were detected with a colloidal staining solution [20 mM CuSO4, 10% (v/v) acetic acid, 45% (v/v) methanol, 0.15% (w/v) Coomassie Brilliant Blue G250 (SERVA Electrophoresis)] and unbound dye was removed by washing in water. Stained proteins were recorded on a Quantum ST4 1100/26MX (Vilber Lourmat) using Quantum-Capt software version 15.12 to estimate transfer efficiency. They are also included as TIFF files. Measurements used for diagrams and statistical analyses in Fig 5A, 5C, 5D, 5E and Fig 6D are deposited in the measurements.xlsx file. Detailed information about secondary antibodies is included in antibodies.pdf. (ZIP) [file pone.0149106.s005.zip › Membrane 1/140213_6_integrin a3_+ruler.Tif]

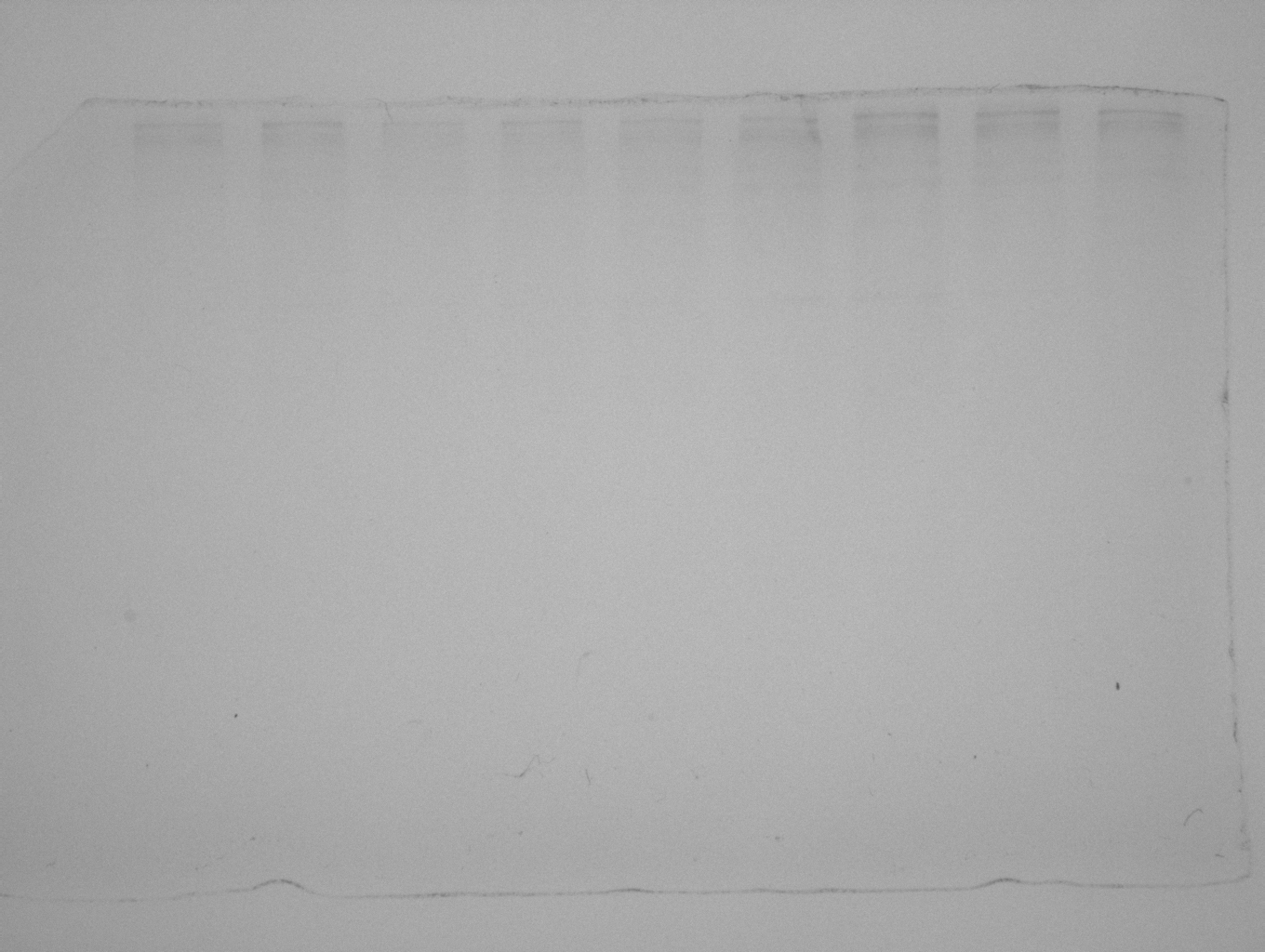

Supplement: S1 Files — Exposures of immunoblot membranes 1, 2, and 3 were used for Fig 2 and exposures of membranes 3, 4 and 5 for S2 Fig. The immunoblot TIFF files are ordered according to stripping steps (1 = before stripping). The positions of the co-electrophoresed size markers were inserted with FusionCapt Advance software version 16.06 on a Fusion-Solo.WL.4M (Vilber Lourmat). The exact details on the ProSieve QuadColor Protein Marker 4.6–300 kDa can be found on the manufacturer’s homepage at http://www.lonza.com/products-services/bio-research/electrophoresis-of-nucleic-acids-and-proteins/protein-electrophoresis/protein-stains-markers/prosieve-protein-colored-and-unstained-markers.aspx. The polypeptides remaining in the SDS-polyacrylamide gels after blotting onto the PVDF membranes were detected with a colloidal staining solution [20 mM CuSO4, 10% (v/v) acetic acid, 45% (v/v) methanol, 0.15% (w/v) Coomassie Brilliant Blue G250 (SERVA Electrophoresis)] and unbound dye was removed by washing in water. Stained proteins were recorded on a Quantum ST4 1100/26MX (Vilber Lourmat) using Quantum-Capt software version 15.12 to estimate transfer efficiency. They are also included as TIFF files. Measurements used for diagrams and statistical analyses in Fig 5A, 5C, 5D, 5E and Fig 6D are deposited in the measurements.xlsx file. Detailed information about secondary antibodies is included in antibodies.pdf. (ZIP) [file pone.0149106.s005.zip › Membrane 1/140213_SDS-PAGE after transfer.Tif]

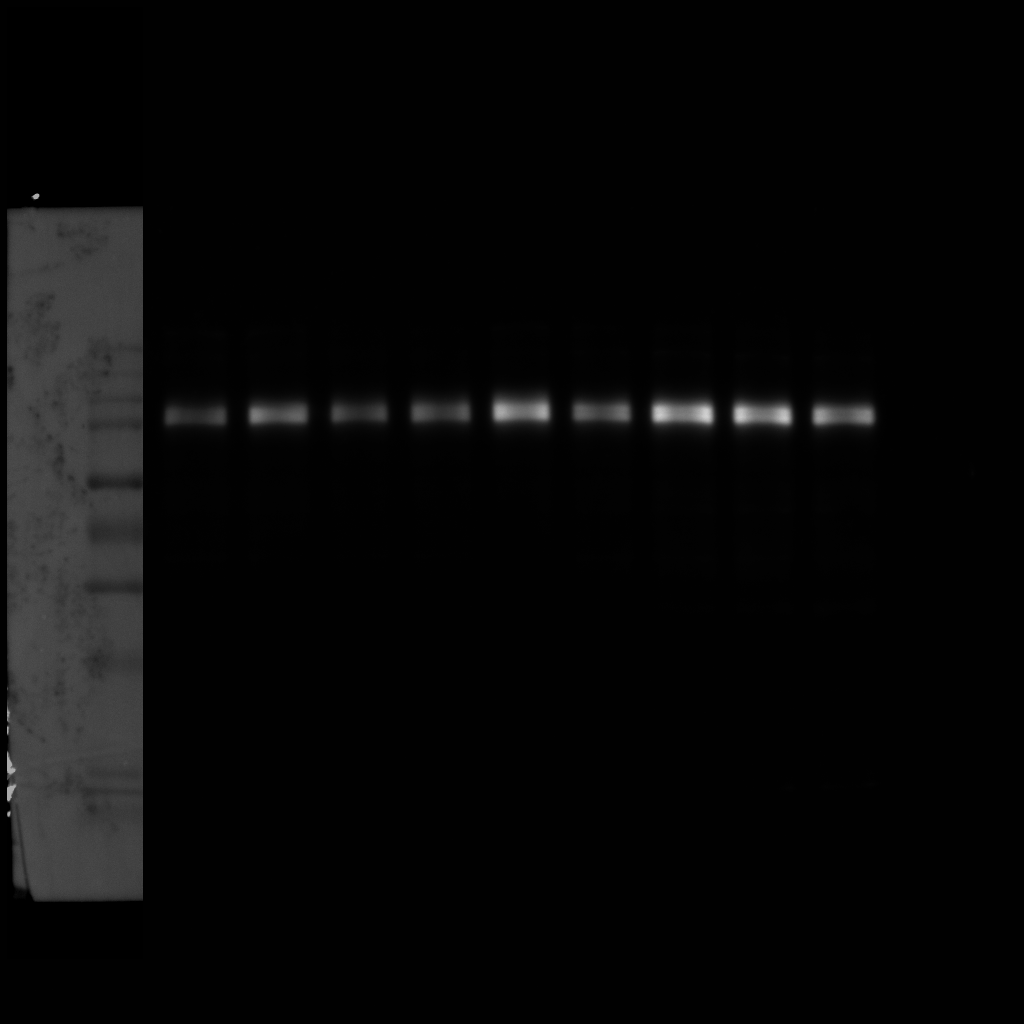

Supplement: S1 Files — Exposures of immunoblot membranes 1, 2, and 3 were used for Fig 2 and exposures of membranes 3, 4 and 5 for S2 Fig. The immunoblot TIFF files are ordered according to stripping steps (1 = before stripping). The positions of the co-electrophoresed size markers were inserted with FusionCapt Advance software version 16.06 on a Fusion-Solo.WL.4M (Vilber Lourmat). The exact details on the ProSieve QuadColor Protein Marker 4.6–300 kDa can be found on the manufacturer’s homepage at http://www.lonza.com/products-services/bio-research/electrophoresis-of-nucleic-acids-and-proteins/protein-electrophoresis/protein-stains-markers/prosieve-protein-colored-and-unstained-markers.aspx. The polypeptides remaining in the SDS-polyacrylamide gels after blotting onto the PVDF membranes were detected with a colloidal staining solution [20 mM CuSO4, 10% (v/v) acetic acid, 45% (v/v) methanol, 0.15% (w/v) Coomassie Brilliant Blue G250 (SERVA Electrophoresis)] and unbound dye was removed by washing in water. Stained proteins were recorded on a Quantum ST4 1100/26MX (Vilber Lourmat) using Quantum-Capt software version 15.12 to estimate transfer efficiency. They are also included as TIFF files. Measurements used for diagrams and statistical analyses in Fig 5A, 5C, 5D, 5E and Fig 6D are deposited in the measurements.xlsx file. Detailed information about secondary antibodies is included in antibodies.pdf. (ZIP) [file pone.0149106.s005.zip › Membrane 2/140211_2_integrin a5_2x binning_+ruler.Tif]

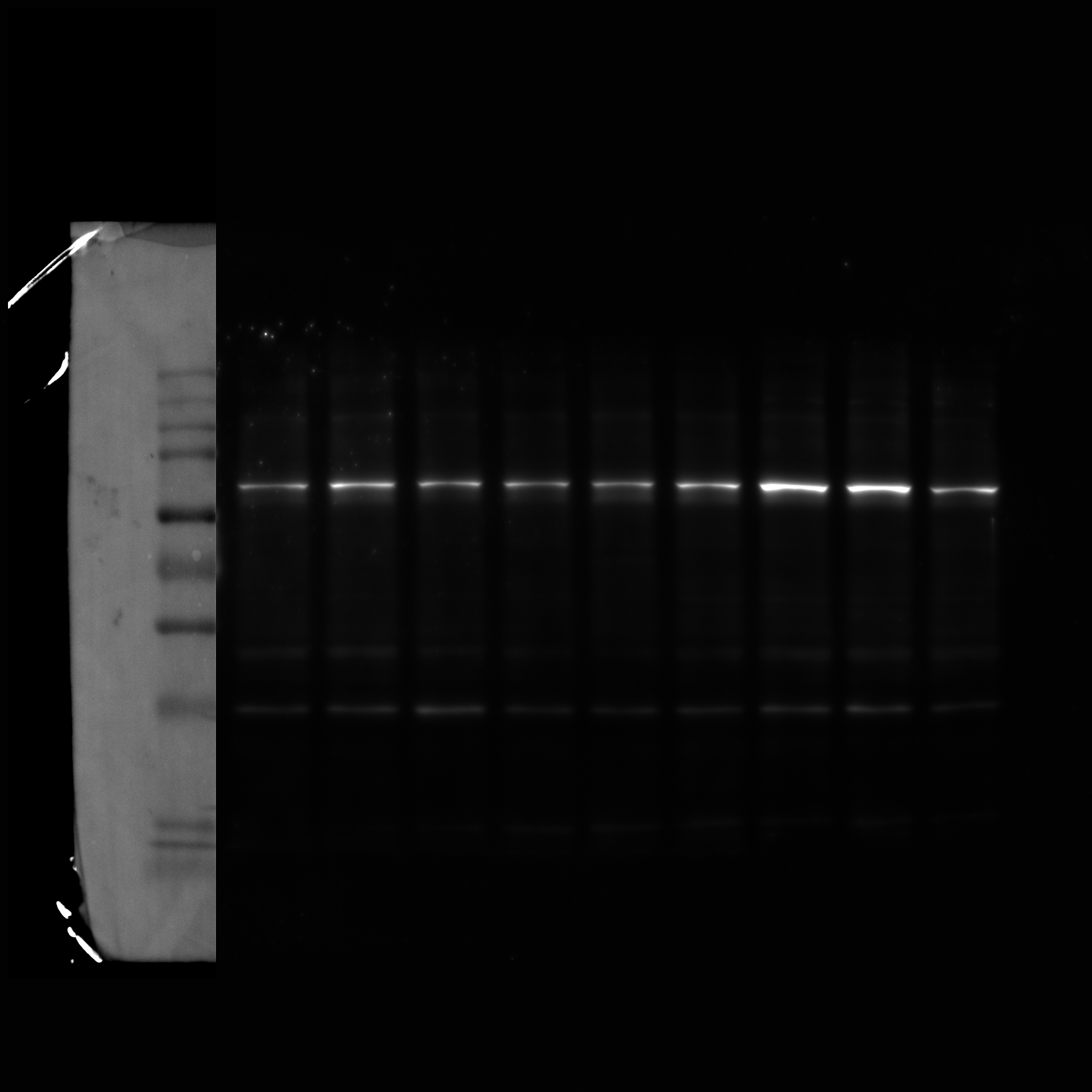

Supplement: S1 Files — Exposures of immunoblot membranes 1, 2, and 3 were used for Fig 2 and exposures of membranes 3, 4 and 5 for S2 Fig. The immunoblot TIFF files are ordered according to stripping steps (1 = before stripping). The positions of the co-electrophoresed size markers were inserted with FusionCapt Advance software version 16.06 on a Fusion-Solo.WL.4M (Vilber Lourmat). The exact details on the ProSieve QuadColor Protein Marker 4.6–300 kDa can be found on the manufacturer’s homepage at http://www.lonza.com/products-services/bio-research/electrophoresis-of-nucleic-acids-and-proteins/protein-electrophoresis/protein-stains-markers/prosieve-protein-colored-and-unstained-markers.aspx. The polypeptides remaining in the SDS-polyacrylamide gels after blotting onto the PVDF membranes were detected with a colloidal staining solution [20 mM CuSO4, 10% (v/v) acetic acid, 45% (v/v) methanol, 0.15% (w/v) Coomassie Brilliant Blue G250 (SERVA Electrophoresis)] and unbound dye was removed by washing in water. Stained proteins were recorded on a Quantum ST4 1100/26MX (Vilber Lourmat) using Quantum-Capt software version 15.12 to estimate transfer efficiency. They are also included as TIFF files. Measurements used for diagrams and statistical analyses in Fig 5A, 5C, 5D, 5E and Fig 6D are deposited in the measurements.xlsx file. Detailed information about secondary antibodies is included in antibodies.pdf. (ZIP) [file pone.0149106.s005.zip › Membrane 2/140211_3_integrin b5_+ruler.Tif]

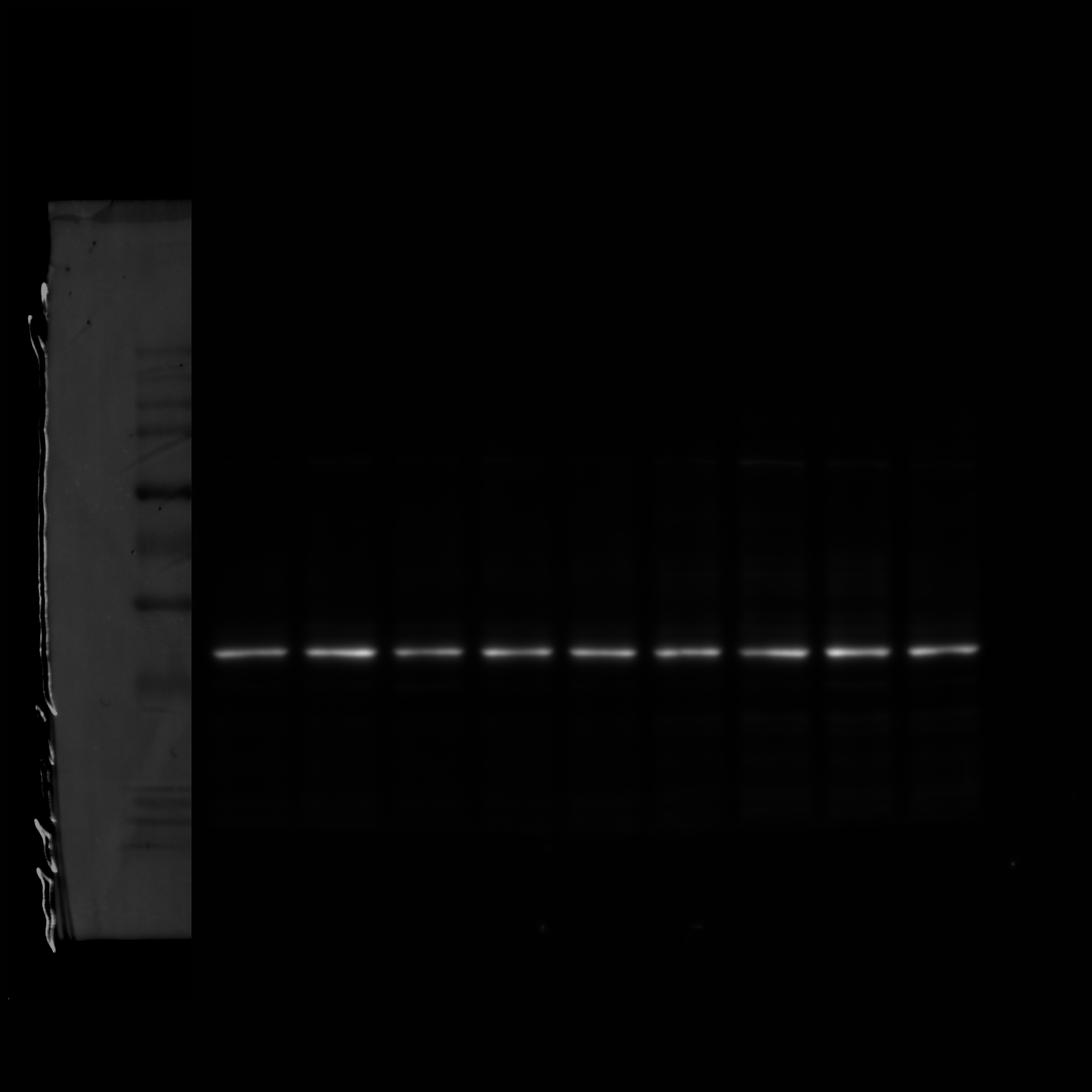

Supplement: S1 Files — Exposures of immunoblot membranes 1, 2, and 3 were used for Fig 2 and exposures of membranes 3, 4 and 5 for S2 Fig. The immunoblot TIFF files are ordered according to stripping steps (1 = before stripping). The positions of the co-electrophoresed size markers were inserted with FusionCapt Advance software version 16.06 on a Fusion-Solo.WL.4M (Vilber Lourmat). The exact details on the ProSieve QuadColor Protein Marker 4.6–300 kDa can be found on the manufacturer’s homepage at http://www.lonza.com/products-services/bio-research/electrophoresis-of-nucleic-acids-and-proteins/protein-electrophoresis/protein-stains-markers/prosieve-protein-colored-and-unstained-markers.aspx. The polypeptides remaining in the SDS-polyacrylamide gels after blotting onto the PVDF membranes were detected with a colloidal staining solution [20 mM CuSO4, 10% (v/v) acetic acid, 45% (v/v) methanol, 0.15% (w/v) Coomassie Brilliant Blue G250 (SERVA Electrophoresis)] and unbound dye was removed by washing in water. Stained proteins were recorded on a Quantum ST4 1100/26MX (Vilber Lourmat) using Quantum-Capt software version 15.12 to estimate transfer efficiency. They are also included as TIFF files. Measurements used for diagrams and statistical analyses in Fig 5A, 5C, 5D, 5E and Fig 6D are deposited in the measurements.xlsx file. Detailed information about secondary antibodies is included in antibodies.pdf. (ZIP) [file pone.0149106.s005.zip › Membrane 2/140211_4_actin_+ruler.Tif]

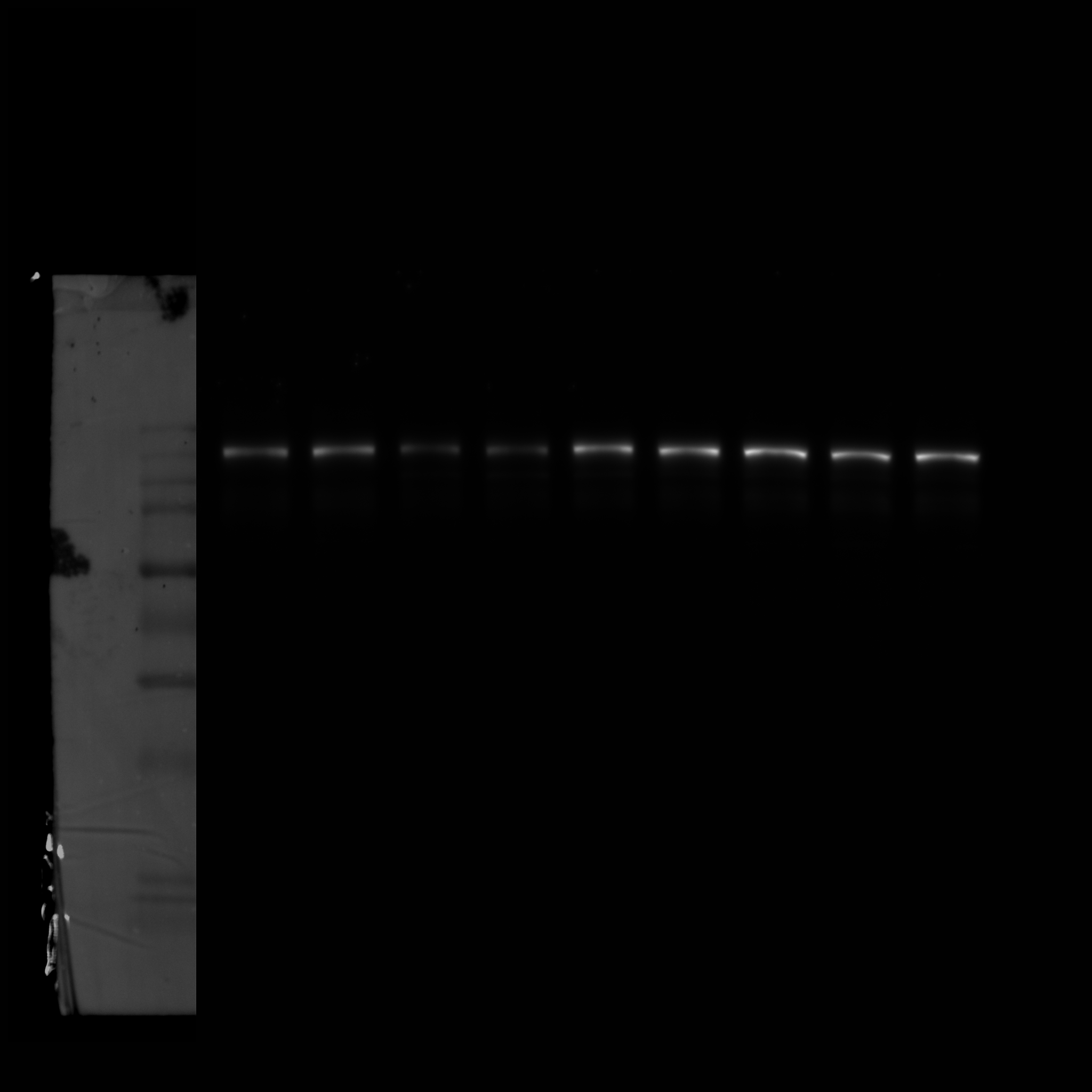

Supplement: S1 Files — Exposures of immunoblot membranes 1, 2, and 3 were used for Fig 2 and exposures of membranes 3, 4 and 5 for S2 Fig. The immunoblot TIFF files are ordered according to stripping steps (1 = before stripping). The positions of the co-electrophoresed size markers were inserted with FusionCapt Advance software version 16.06 on a Fusion-Solo.WL.4M (Vilber Lourmat). The exact details on the ProSieve QuadColor Protein Marker 4.6–300 kDa can be found on the manufacturer’s homepage at http://www.lonza.com/products-services/bio-research/electrophoresis-of-nucleic-acids-and-proteins/protein-electrophoresis/protein-stains-markers/prosieve-protein-colored-and-unstained-markers.aspx. The polypeptides remaining in the SDS-polyacrylamide gels after blotting onto the PVDF membranes were detected with a colloidal staining solution [20 mM CuSO4, 10% (v/v) acetic acid, 45% (v/v) methanol, 0.15% (w/v) Coomassie Brilliant Blue G250 (SERVA Electrophoresis)] and unbound dye was removed by washing in water. Stained proteins were recorded on a Quantum ST4 1100/26MX (Vilber Lourmat) using Quantum-Capt software version 15.12 to estimate transfer efficiency. They are also included as TIFF files. Measurements used for diagrams and statistical analyses in Fig 5A, 5C, 5D, 5E and Fig 6D are deposited in the measurements.xlsx file. Detailed information about secondary antibodies is included in antibodies.pdf. (ZIP) [file pone.0149106.s005.zip › Membrane 2/140211_5_integrin b4_+ruler.Tif]

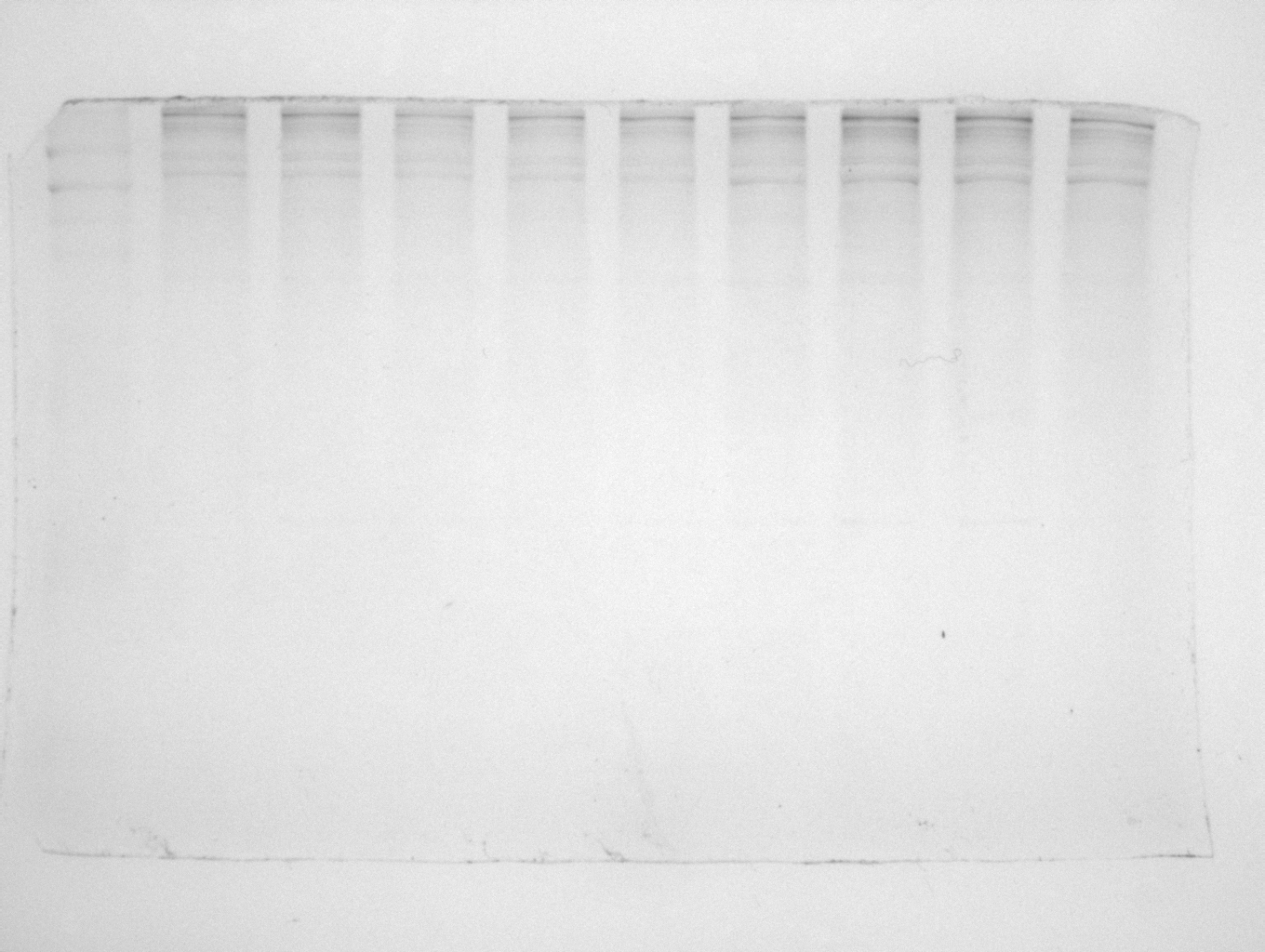

Supplement: S1 Files — Exposures of immunoblot membranes 1, 2, and 3 were used for Fig 2 and exposures of membranes 3, 4 and 5 for S2 Fig. The immunoblot TIFF files are ordered according to stripping steps (1 = before stripping). The positions of the co-electrophoresed size markers were inserted with FusionCapt Advance software version 16.06 on a Fusion-Solo.WL.4M (Vilber Lourmat). The exact details on the ProSieve QuadColor Protein Marker 4.6–300 kDa can be found on the manufacturer’s homepage at http://www.lonza.com/products-services/bio-research/electrophoresis-of-nucleic-acids-and-proteins/protein-electrophoresis/protein-stains-markers/prosieve-protein-colored-and-unstained-markers.aspx. The polypeptides remaining in the SDS-polyacrylamide gels after blotting onto the PVDF membranes were detected with a colloidal staining solution [20 mM CuSO4, 10% (v/v) acetic acid, 45% (v/v) methanol, 0.15% (w/v) Coomassie Brilliant Blue G250 (SERVA Electrophoresis)] and unbound dye was removed by washing in water. Stained proteins were recorded on a Quantum ST4 1100/26MX (Vilber Lourmat) using Quantum-Capt software version 15.12 to estimate transfer efficiency. They are also included as TIFF files. Measurements used for diagrams and statistical analyses in Fig 5A, 5C, 5D, 5E and Fig 6D are deposited in the measurements.xlsx file. Detailed information about secondary antibodies is included in antibodies.pdf. (ZIP) [file pone.0149106.s005.zip › Membrane 2/140211_SDS-PAGE after transfer.Tif]

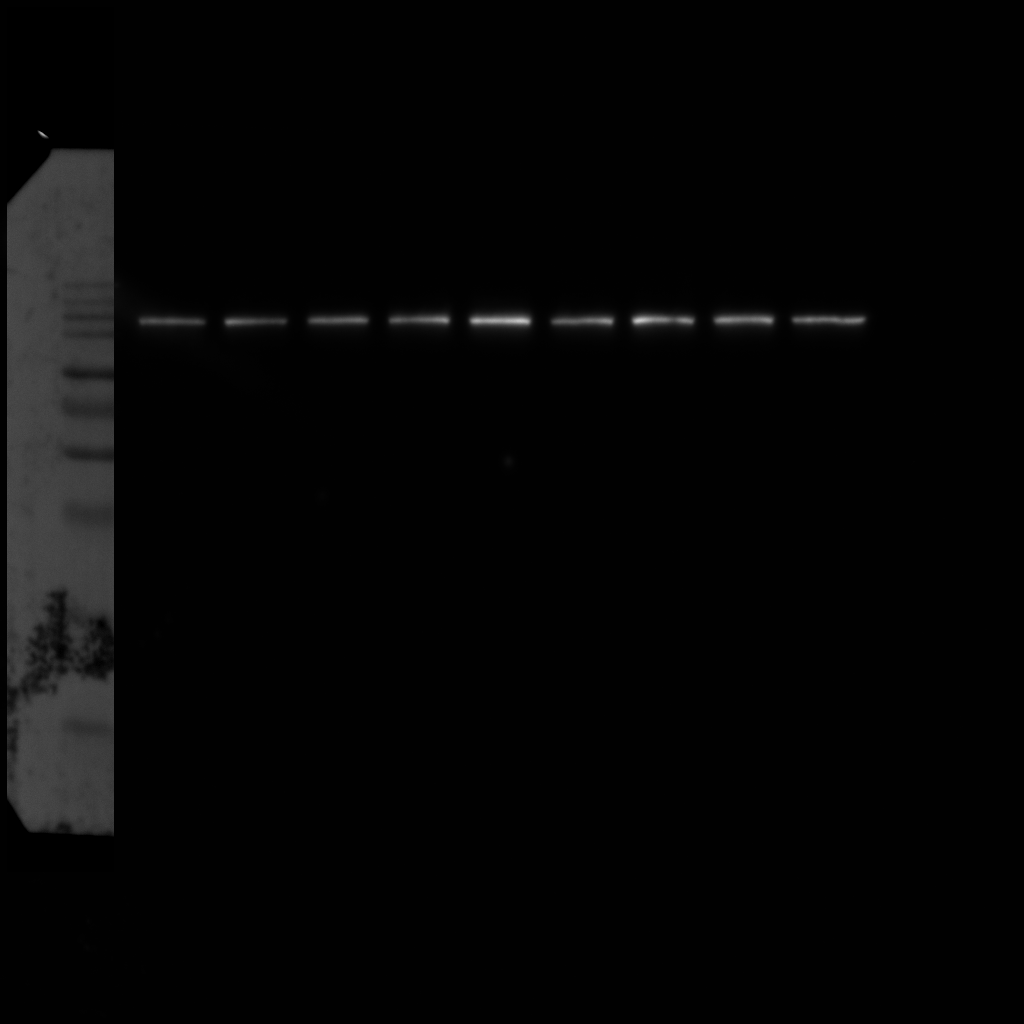

Supplement: S1 Files — Exposures of immunoblot membranes 1, 2, and 3 were used for Fig 2 and exposures of membranes 3, 4 and 5 for S2 Fig. The immunoblot TIFF files are ordered according to stripping steps (1 = before stripping). The positions of the co-electrophoresed size markers were inserted with FusionCapt Advance software version 16.06 on a Fusion-Solo.WL.4M (Vilber Lourmat). The exact details on the ProSieve QuadColor Protein Marker 4.6–300 kDa can be found on the manufacturer’s homepage at http://www.lonza.com/products-services/bio-research/electrophoresis-of-nucleic-acids-and-proteins/protein-electrophoresis/protein-stains-markers/prosieve-protein-colored-and-unstained-markers.aspx. The polypeptides remaining in the SDS-polyacrylamide gels after blotting onto the PVDF membranes were detected with a colloidal staining solution [20 mM CuSO4, 10% (v/v) acetic acid, 45% (v/v) methanol, 0.15% (w/v) Coomassie Brilliant Blue G250 (SERVA Electrophoresis)] and unbound dye was removed by washing in water. Stained proteins were recorded on a Quantum ST4 1100/26MX (Vilber Lourmat) using Quantum-Capt software version 15.12 to estimate transfer efficiency. They are also included as TIFF files. Measurements used for diagrams and statistical analyses in Fig 5A, 5C, 5D, 5E and Fig 6D are deposited in the measurements.xlsx file. Detailed information about secondary antibodies is included in antibodies.pdf. (ZIP) [file pone.0149106.s005.zip › Membrane 3/140305_1_integrin a2_2x binning_+ruler.Tif]

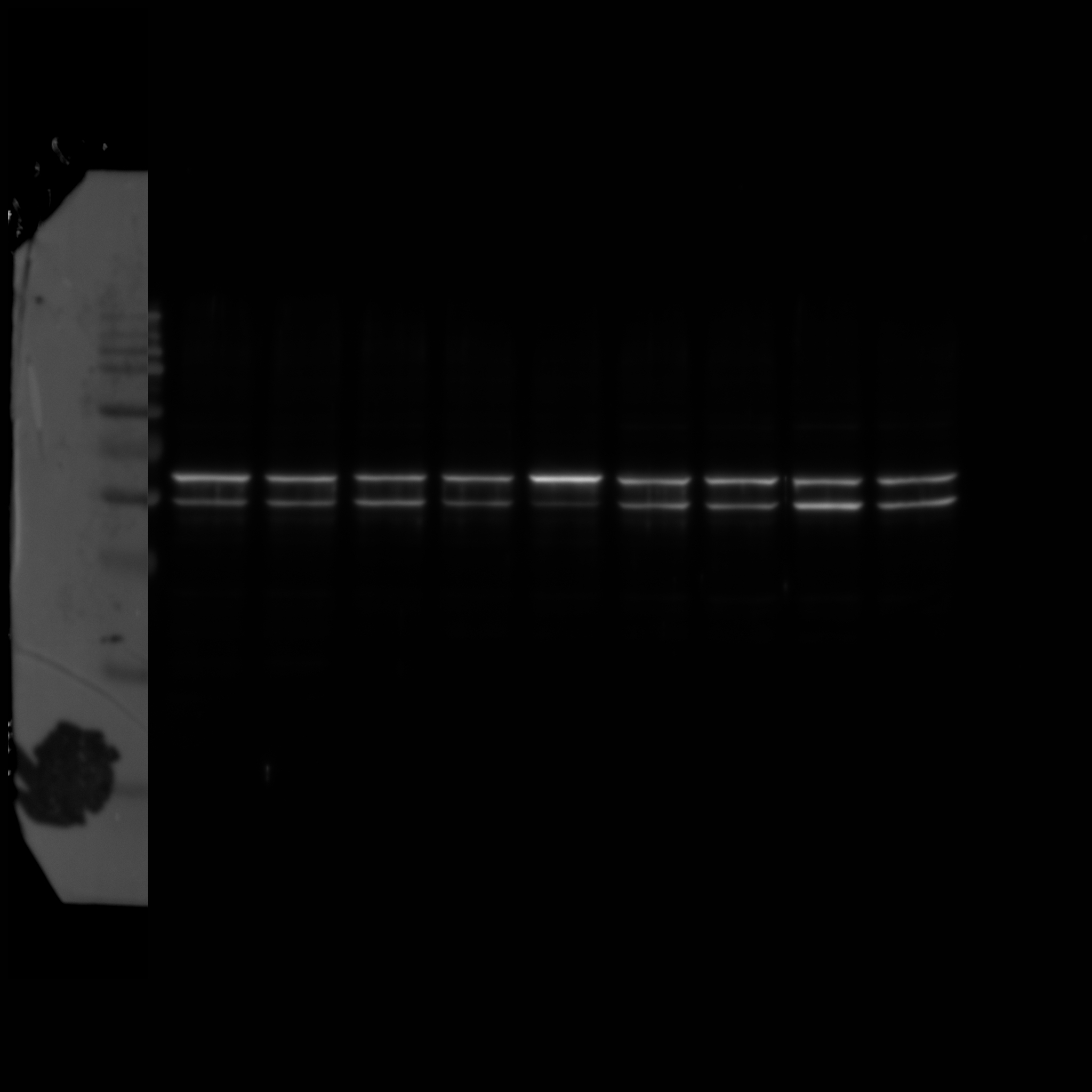

Supplement: S1 Files — Exposures of immunoblot membranes 1, 2, and 3 were used for Fig 2 and exposures of membranes 3, 4 and 5 for S2 Fig. The immunoblot TIFF files are ordered according to stripping steps (1 = before stripping). The positions of the co-electrophoresed size markers were inserted with FusionCapt Advance software version 16.06 on a Fusion-Solo.WL.4M (Vilber Lourmat). The exact details on the ProSieve QuadColor Protein Marker 4.6–300 kDa can be found on the manufacturer’s homepage at http://www.lonza.com/products-services/bio-research/electrophoresis-of-nucleic-acids-and-proteins/protein-electrophoresis/protein-stains-markers/prosieve-protein-colored-and-unstained-markers.aspx. The polypeptides remaining in the SDS-polyacrylamide gels after blotting onto the PVDF membranes were detected with a colloidal staining solution [20 mM CuSO4, 10% (v/v) acetic acid, 45% (v/v) methanol, 0.15% (w/v) Coomassie Brilliant Blue G250 (SERVA Electrophoresis)] and unbound dye was removed by washing in water. Stained proteins were recorded on a Quantum ST4 1100/26MX (Vilber Lourmat) using Quantum-Capt software version 15.12 to estimate transfer efficiency. They are also included as TIFF files. Measurements used for diagrams and statistical analyses in Fig 5A, 5C, 5D, 5E and Fig 6D are deposited in the measurements.xlsx file. Detailed information about secondary antibodies is included in antibodies.pdf. (ZIP) [file pone.0149106.s005.zip › Membrane 3/140305_2_keratin 5_+ruler.Tif]

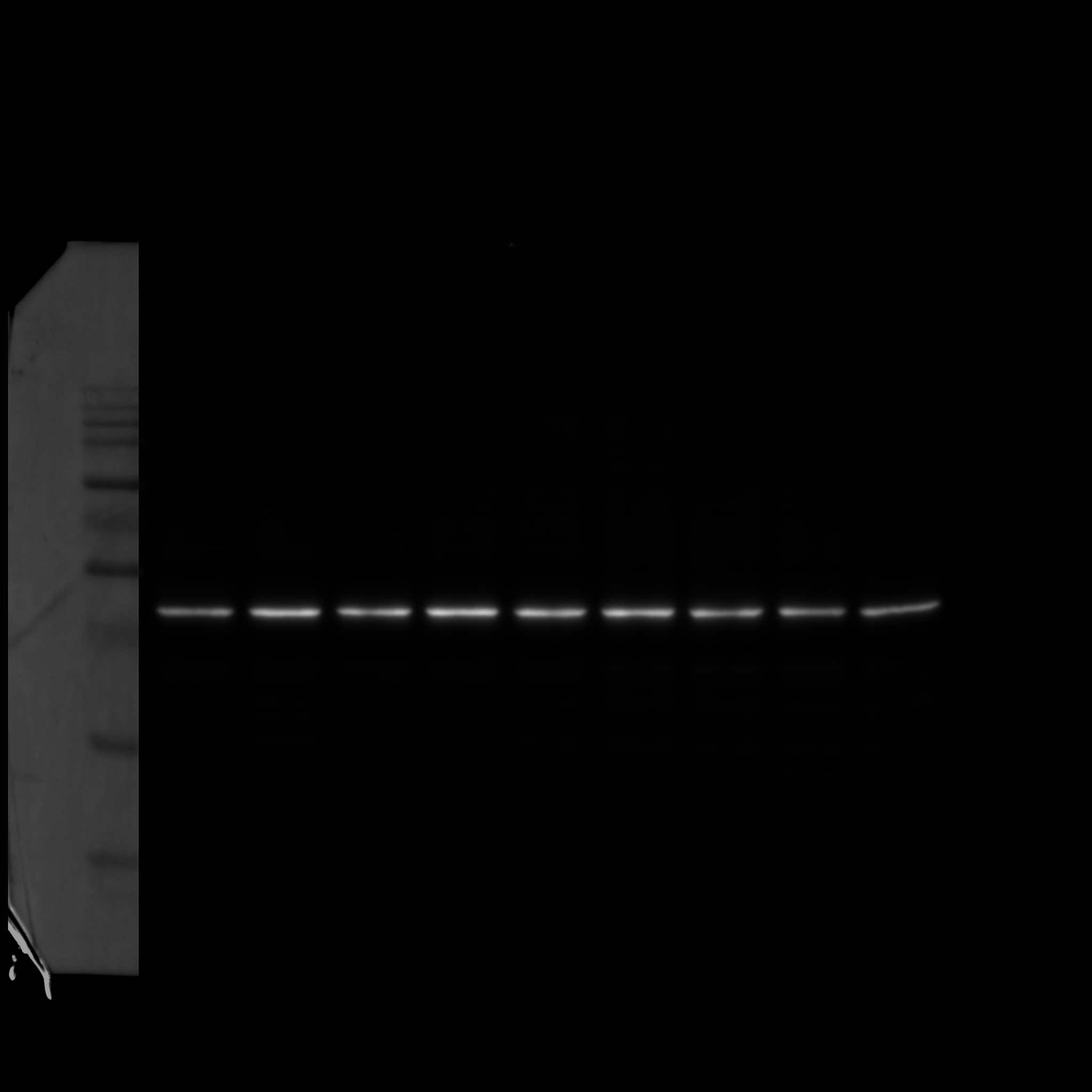

Supplement: S1 Files — Exposures of immunoblot membranes 1, 2, and 3 were used for Fig 2 and exposures of membranes 3, 4 and 5 for S2 Fig. The immunoblot TIFF files are ordered according to stripping steps (1 = before stripping). The positions of the co-electrophoresed size markers were inserted with FusionCapt Advance software version 16.06 on a Fusion-Solo.WL.4M (Vilber Lourmat). The exact details on the ProSieve QuadColor Protein Marker 4.6–300 kDa can be found on the manufacturer’s homepage at http://www.lonza.com/products-services/bio-research/electrophoresis-of-nucleic-acids-and-proteins/protein-electrophoresis/protein-stains-markers/prosieve-protein-colored-and-unstained-markers.aspx. The polypeptides remaining in the SDS-polyacrylamide gels after blotting onto the PVDF membranes were detected with a colloidal staining solution [20 mM CuSO4, 10% (v/v) acetic acid, 45% (v/v) methanol, 0.15% (w/v) Coomassie Brilliant Blue G250 (SERVA Electrophoresis)] and unbound dye was removed by washing in water. Stained proteins were recorded on a Quantum ST4 1100/26MX (Vilber Lourmat) using Quantum-Capt software version 15.12 to estimate transfer efficiency. They are also included as TIFF files. Measurements used for diagrams and statistical analyses in Fig 5A, 5C, 5D, 5E and Fig 6D are deposited in the measurements.xlsx file. Detailed information about secondary antibodies is included in antibodies.pdf. (ZIP) [file pone.0149106.s005.zip › Membrane 3/140305_4_actin_+ruler.Tif]

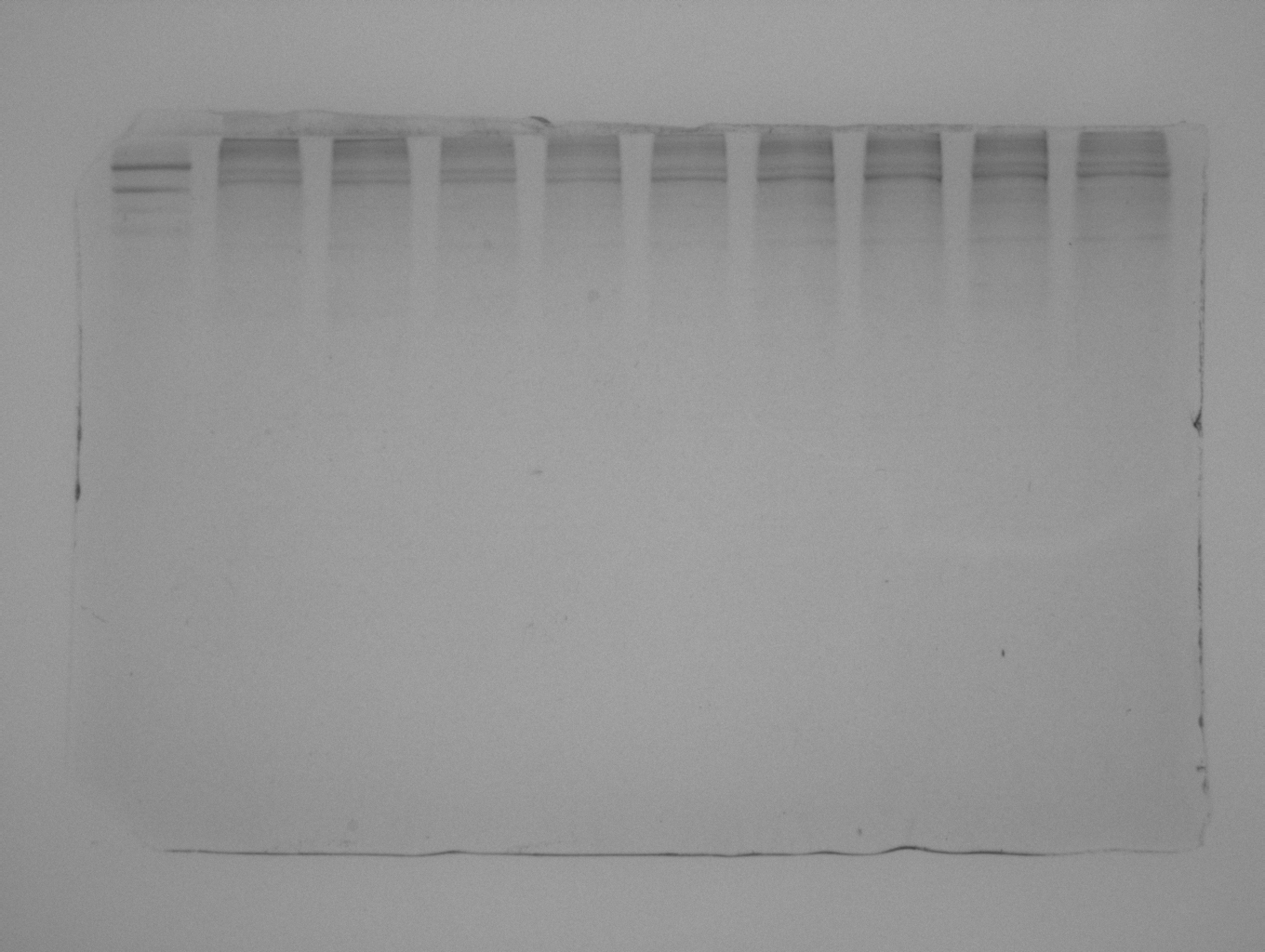

Supplement: S1 Files — Exposures of immunoblot membranes 1, 2, and 3 were used for Fig 2 and exposures of membranes 3, 4 and 5 for S2 Fig. The immunoblot TIFF files are ordered according to stripping steps (1 = before stripping). The positions of the co-electrophoresed size markers were inserted with FusionCapt Advance software version 16.06 on a Fusion-Solo.WL.4M (Vilber Lourmat). The exact details on the ProSieve QuadColor Protein Marker 4.6–300 kDa can be found on the manufacturer’s homepage at http://www.lonza.com/products-services/bio-research/electrophoresis-of-nucleic-acids-and-proteins/protein-electrophoresis/protein-stains-markers/prosieve-protein-colored-and-unstained-markers.aspx. The polypeptides remaining in the SDS-polyacrylamide gels after blotting onto the PVDF membranes were detected with a colloidal staining solution [20 mM CuSO4, 10% (v/v) acetic acid, 45% (v/v) methanol, 0.15% (w/v) Coomassie Brilliant Blue G250 (SERVA Electrophoresis)] and unbound dye was removed by washing in water. Stained proteins were recorded on a Quantum ST4 1100/26MX (Vilber Lourmat) using Quantum-Capt software version 15.12 to estimate transfer efficiency. They are also included as TIFF files. Measurements used for diagrams and statistical analyses in Fig 5A, 5C, 5D, 5E and Fig 6D are deposited in the measurements.xlsx file. Detailed information about secondary antibodies is included in antibodies.pdf. (ZIP) [file pone.0149106.s005.zip › Membrane 3/140305_SDS-PAGE after transfer.Tif]

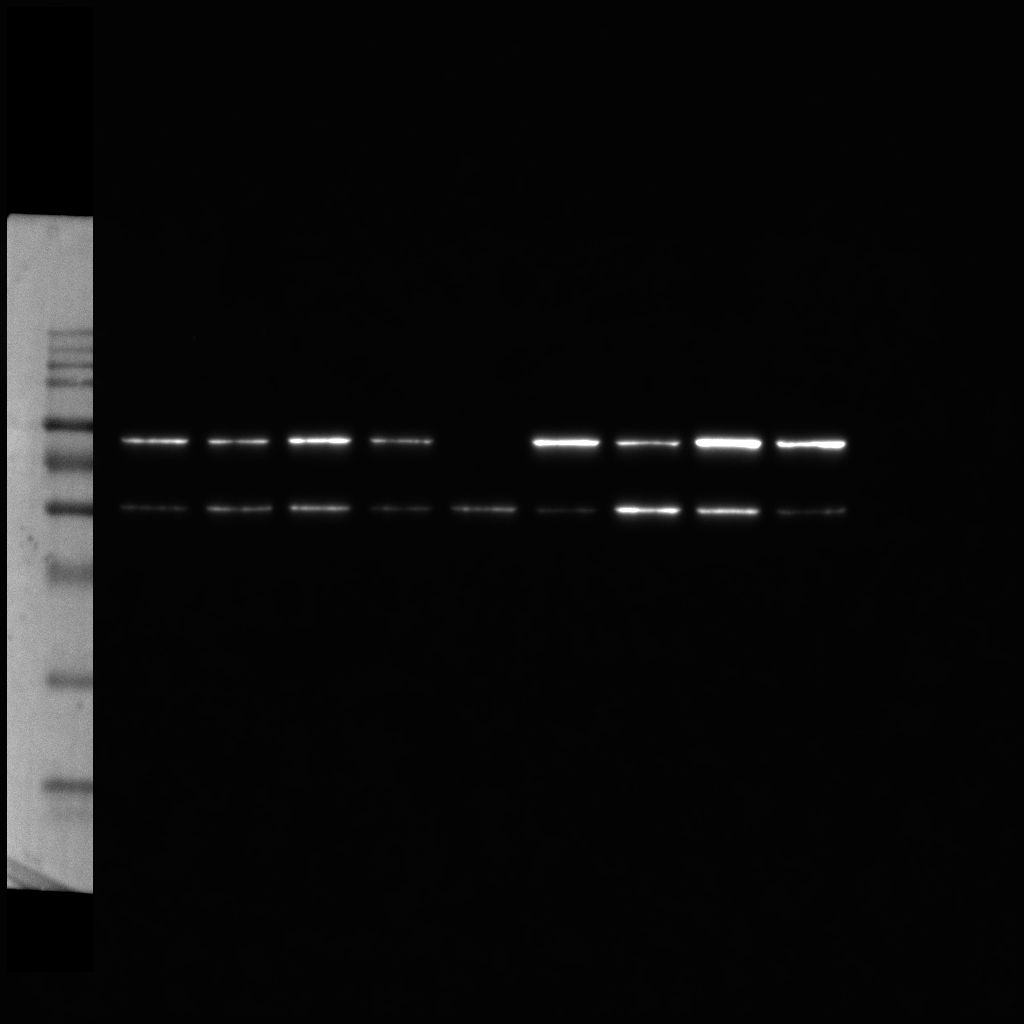

Supplement: S1 Files — Exposures of immunoblot membranes 1, 2, and 3 were used for Fig 2 and exposures of membranes 3, 4 and 5 for S2 Fig. The immunoblot TIFF files are ordered according to stripping steps (1 = before stripping). The positions of the co-electrophoresed size markers were inserted with FusionCapt Advance software version 16.06 on a Fusion-Solo.WL.4M (Vilber Lourmat). The exact details on the ProSieve QuadColor Protein Marker 4.6–300 kDa can be found on the manufacturer’s homepage at http://www.lonza.com/products-services/bio-research/electrophoresis-of-nucleic-acids-and-proteins/protein-electrophoresis/protein-stains-markers/prosieve-protein-colored-and-unstained-markers.aspx. The polypeptides remaining in the SDS-polyacrylamide gels after blotting onto the PVDF membranes were detected with a colloidal staining solution [20 mM CuSO4, 10% (v/v) acetic acid, 45% (v/v) methanol, 0.15% (w/v) Coomassie Brilliant Blue G250 (SERVA Electrophoresis)] and unbound dye was removed by washing in water. Stained proteins were recorded on a Quantum ST4 1100/26MX (Vilber Lourmat) using Quantum-Capt software version 15.12 to estimate transfer efficiency. They are also included as TIFF files. Measurements used for diagrams and statistical analyses in Fig 5A, 5C, 5D, 5E and Fig 6D are deposited in the measurements.xlsx file. Detailed information about secondary antibodies is included in antibodies.pdf. (ZIP) [file pone.0149106.s005.zip › Membrane 4/140305_1_keratin 13_binning 2x_+ruler.Tif]

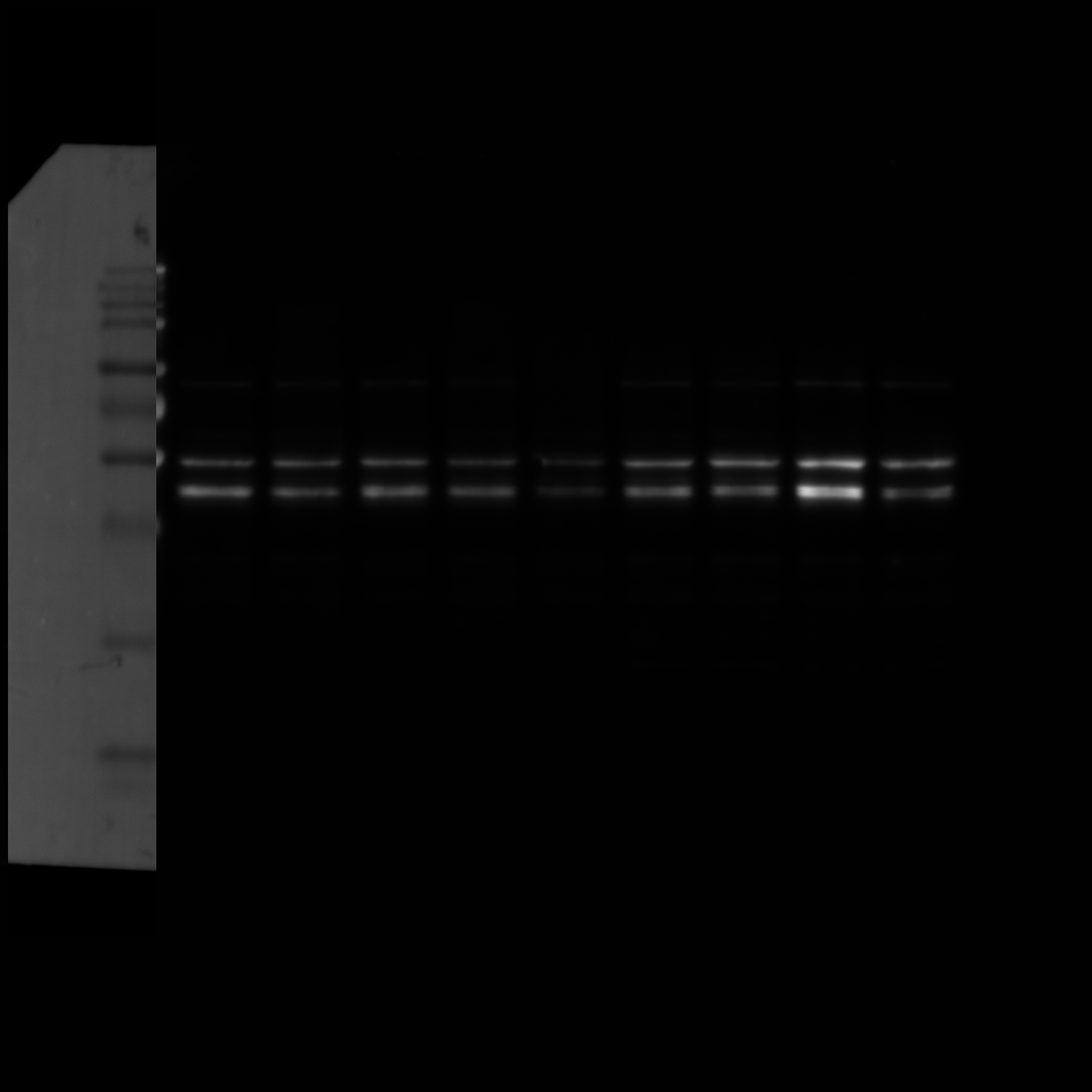

Supplement: S1 Files — Exposures of immunoblot membranes 1, 2, and 3 were used for Fig 2 and exposures of membranes 3, 4 and 5 for S2 Fig. The immunoblot TIFF files are ordered according to stripping steps (1 = before stripping). The positions of the co-electrophoresed size markers were inserted with FusionCapt Advance software version 16.06 on a Fusion-Solo.WL.4M (Vilber Lourmat). The exact details on the ProSieve QuadColor Protein Marker 4.6–300 kDa can be found on the manufacturer’s homepage at http://www.lonza.com/products-services/bio-research/electrophoresis-of-nucleic-acids-and-proteins/protein-electrophoresis/protein-stains-markers/prosieve-protein-colored-and-unstained-markers.aspx. The polypeptides remaining in the SDS-polyacrylamide gels after blotting onto the PVDF membranes were detected with a colloidal staining solution [20 mM CuSO4, 10% (v/v) acetic acid, 45% (v/v) methanol, 0.15% (w/v) Coomassie Brilliant Blue G250 (SERVA Electrophoresis)] and unbound dye was removed by washing in water. Stained proteins were recorded on a Quantum ST4 1100/26MX (Vilber Lourmat) using Quantum-Capt software version 15.12 to estimate transfer efficiency. They are also included as TIFF files. Measurements used for diagrams and statistical analyses in Fig 5A, 5C, 5D, 5E and Fig 6D are deposited in the measurements.xlsx file. Detailed information about secondary antibodies is included in antibodies.pdf. (ZIP) [file pone.0149106.s005.zip › Membrane 4/140305_2_keratins 8 and 18_+ruler.Tif]

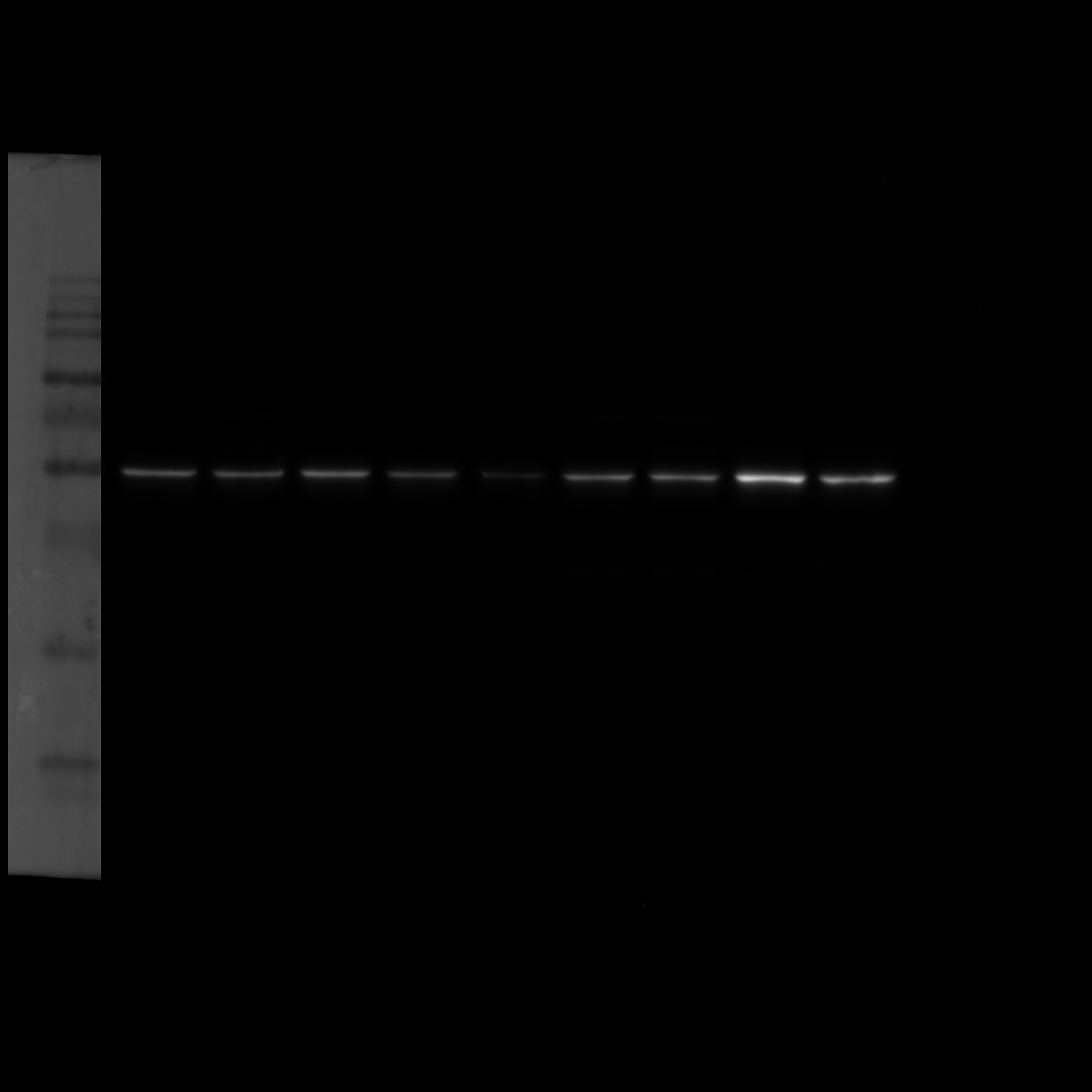

Supplement: S1 Files — Exposures of immunoblot membranes 1, 2, and 3 were used for Fig 2 and exposures of membranes 3, 4 and 5 for S2 Fig. The immunoblot TIFF files are ordered according to stripping steps (1 = before stripping). The positions of the co-electrophoresed size markers were inserted with FusionCapt Advance software version 16.06 on a Fusion-Solo.WL.4M (Vilber Lourmat). The exact details on the ProSieve QuadColor Protein Marker 4.6–300 kDa can be found on the manufacturer’s homepage at http://www.lonza.com/products-services/bio-research/electrophoresis-of-nucleic-acids-and-proteins/protein-electrophoresis/protein-stains-markers/prosieve-protein-colored-and-unstained-markers.aspx. The polypeptides remaining in the SDS-polyacrylamide gels after blotting onto the PVDF membranes were detected with a colloidal staining solution [20 mM CuSO4, 10% (v/v) acetic acid, 45% (v/v) methanol, 0.15% (w/v) Coomassie Brilliant Blue G250 (SERVA Electrophoresis)] and unbound dye was removed by washing in water. Stained proteins were recorded on a Quantum ST4 1100/26MX (Vilber Lourmat) using Quantum-Capt software version 15.12 to estimate transfer efficiency. They are also included as TIFF files. Measurements used for diagrams and statistical analyses in Fig 5A, 5C, 5D, 5E and Fig 6D are deposited in the measurements.xlsx file. Detailed information about secondary antibodies is included in antibodies.pdf. (ZIP) [file pone.0149106.s005.zip › Membrane 4/140305_3_keratin 8_+ruler.Tif]

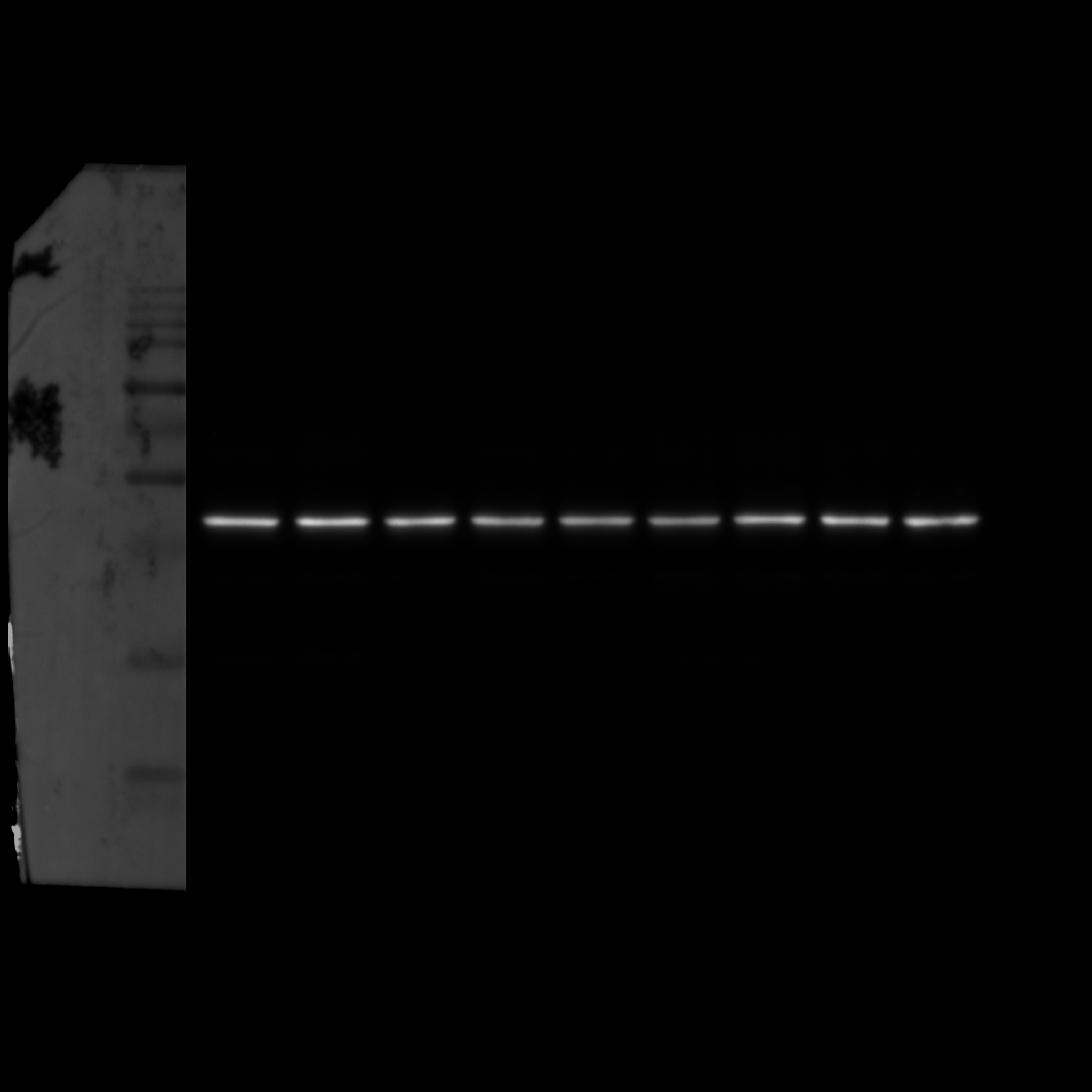

Supplement: S1 Files — Exposures of immunoblot membranes 1, 2, and 3 were used for Fig 2 and exposures of membranes 3, 4 and 5 for S2 Fig. The immunoblot TIFF files are ordered according to stripping steps (1 = before stripping). The positions of the co-electrophoresed size markers were inserted with FusionCapt Advance software version 16.06 on a Fusion-Solo.WL.4M (Vilber Lourmat). The exact details on the ProSieve QuadColor Protein Marker 4.6–300 kDa can be found on the manufacturer’s homepage at http://www.lonza.com/products-services/bio-research/electrophoresis-of-nucleic-acids-and-proteins/protein-electrophoresis/protein-stains-markers/prosieve-protein-colored-and-unstained-markers.aspx. The polypeptides remaining in the SDS-polyacrylamide gels after blotting onto the PVDF membranes were detected with a colloidal staining solution [20 mM CuSO4, 10% (v/v) acetic acid, 45% (v/v) methanol, 0.15% (w/v) Coomassie Brilliant Blue G250 (SERVA Electrophoresis)] and unbound dye was removed by washing in water. Stained proteins were recorded on a Quantum ST4 1100/26MX (Vilber Lourmat) using Quantum-Capt software version 15.12 to estimate transfer efficiency. They are also included as TIFF files. Measurements used for diagrams and statistical analyses in Fig 5A, 5C, 5D, 5E and Fig 6D are deposited in the measurements.xlsx file. Detailed information about secondary antibodies is included in antibodies.pdf. (ZIP) [file pone.0149106.s005.zip › Membrane 4/140305_4_actin_+ruler.Tif]

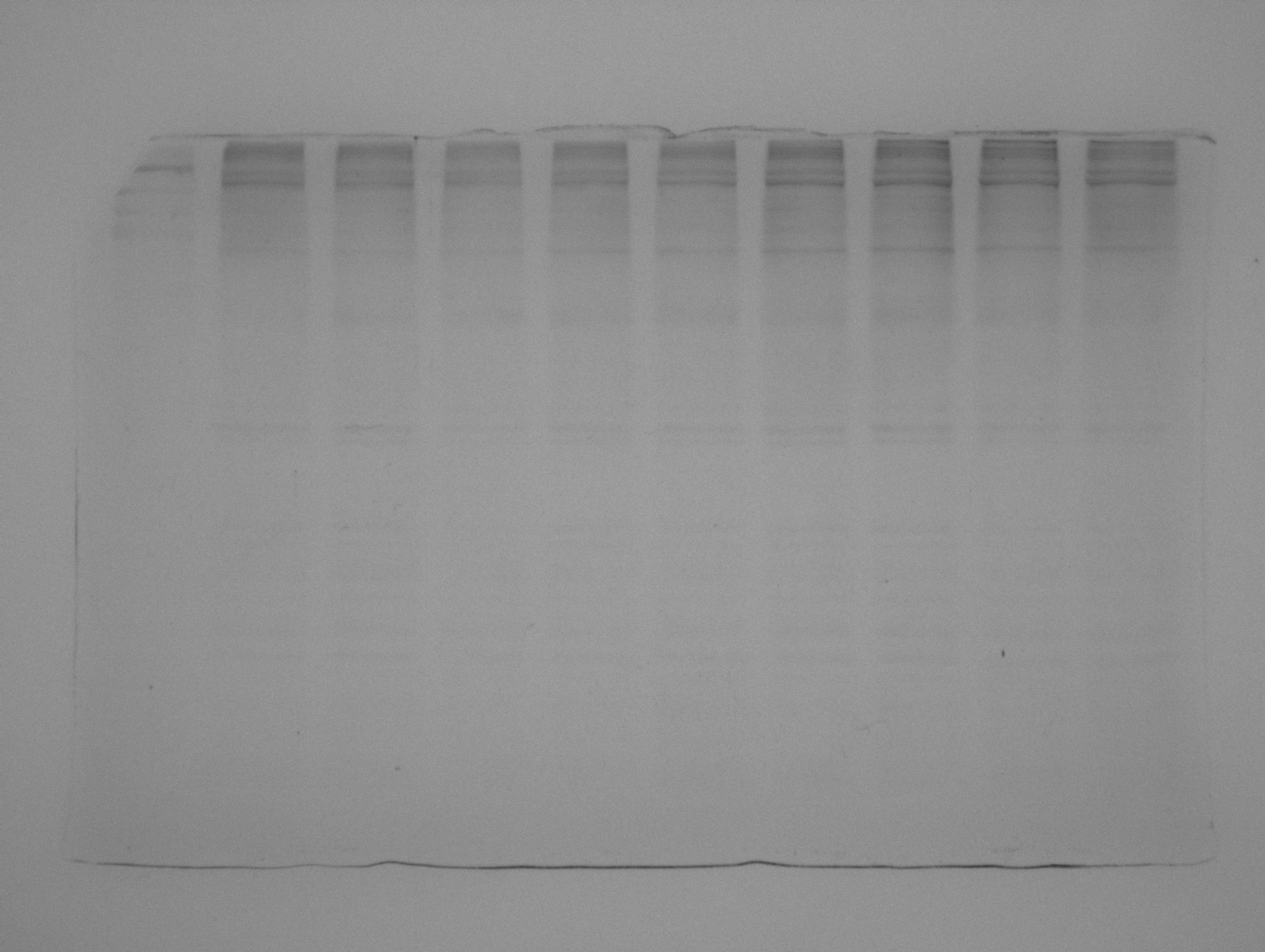

Supplement: S1 Files — Exposures of immunoblot membranes 1, 2, and 3 were used for Fig 2 and exposures of membranes 3, 4 and 5 for S2 Fig. The immunoblot TIFF files are ordered according to stripping steps (1 = before stripping). The positions of the co-electrophoresed size markers were inserted with FusionCapt Advance software version 16.06 on a Fusion-Solo.WL.4M (Vilber Lourmat). The exact details on the ProSieve QuadColor Protein Marker 4.6–300 kDa can be found on the manufacturer’s homepage at http://www.lonza.com/products-services/bio-research/electrophoresis-of-nucleic-acids-and-proteins/protein-electrophoresis/protein-stains-markers/prosieve-protein-colored-and-unstained-markers.aspx. The polypeptides remaining in the SDS-polyacrylamide gels after blotting onto the PVDF membranes were detected with a colloidal staining solution [20 mM CuSO4, 10% (v/v) acetic acid, 45% (v/v) methanol, 0.15% (w/v) Coomassie Brilliant Blue G250 (SERVA Electrophoresis)] and unbound dye was removed by washing in water. Stained proteins were recorded on a Quantum ST4 1100/26MX (Vilber Lourmat) using Quantum-Capt software version 15.12 to estimate transfer efficiency. They are also included as TIFF files. Measurements used for diagrams and statistical analyses in Fig 5A, 5C, 5D, 5E and Fig 6D are deposited in the measurements.xlsx file. Detailed information about secondary antibodies is included in antibodies.pdf. (ZIP) [file pone.0149106.s005.zip › Membrane 4/140305_SDS-PAGE after transfer.Tif]

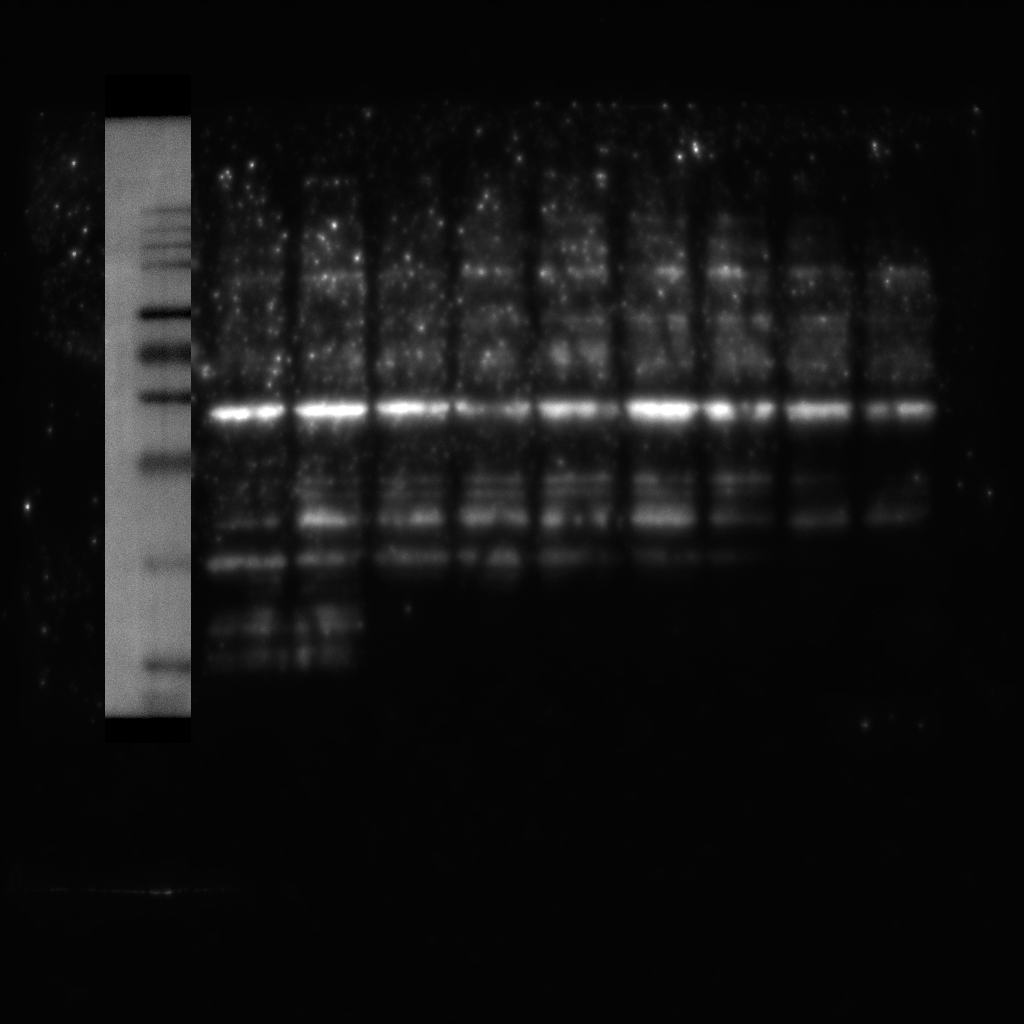

Supplement: S1 Files — Exposures of immunoblot membranes 1, 2, and 3 were used for Fig 2 and exposures of membranes 3, 4 and 5 for S2 Fig. The immunoblot TIFF files are ordered according to stripping steps (1 = before stripping). The positions of the co-electrophoresed size markers were inserted with FusionCapt Advance software version 16.06 on a Fusion-Solo.WL.4M (Vilber Lourmat). The exact details on the ProSieve QuadColor Protein Marker 4.6–300 kDa can be found on the manufacturer’s homepage at http://www.lonza.com/products-services/bio-research/electrophoresis-of-nucleic-acids-and-proteins/protein-electrophoresis/protein-stains-markers/prosieve-protein-colored-and-unstained-markers.aspx. The polypeptides remaining in the SDS-polyacrylamide gels after blotting onto the PVDF membranes were detected with a colloidal staining solution [20 mM CuSO4, 10% (v/v) acetic acid, 45% (v/v) methanol, 0.15% (w/v) Coomassie Brilliant Blue G250 (SERVA Electrophoresis)] and unbound dye was removed by washing in water. Stained proteins were recorded on a Quantum ST4 1100/26MX (Vilber Lourmat) using Quantum-Capt software version 15.12 to estimate transfer efficiency. They are also included as TIFF files. Measurements used for diagrams and statistical analyses in Fig 5A, 5C, 5D, 5E and Fig 6D are deposited in the measurements.xlsx file. Detailed information about secondary antibodies is included in antibodies.pdf. (ZIP) [file pone.0149106.s005.zip › Membrane 5/151220_1_keratin 17_binning 2x_+ruler.Tif]

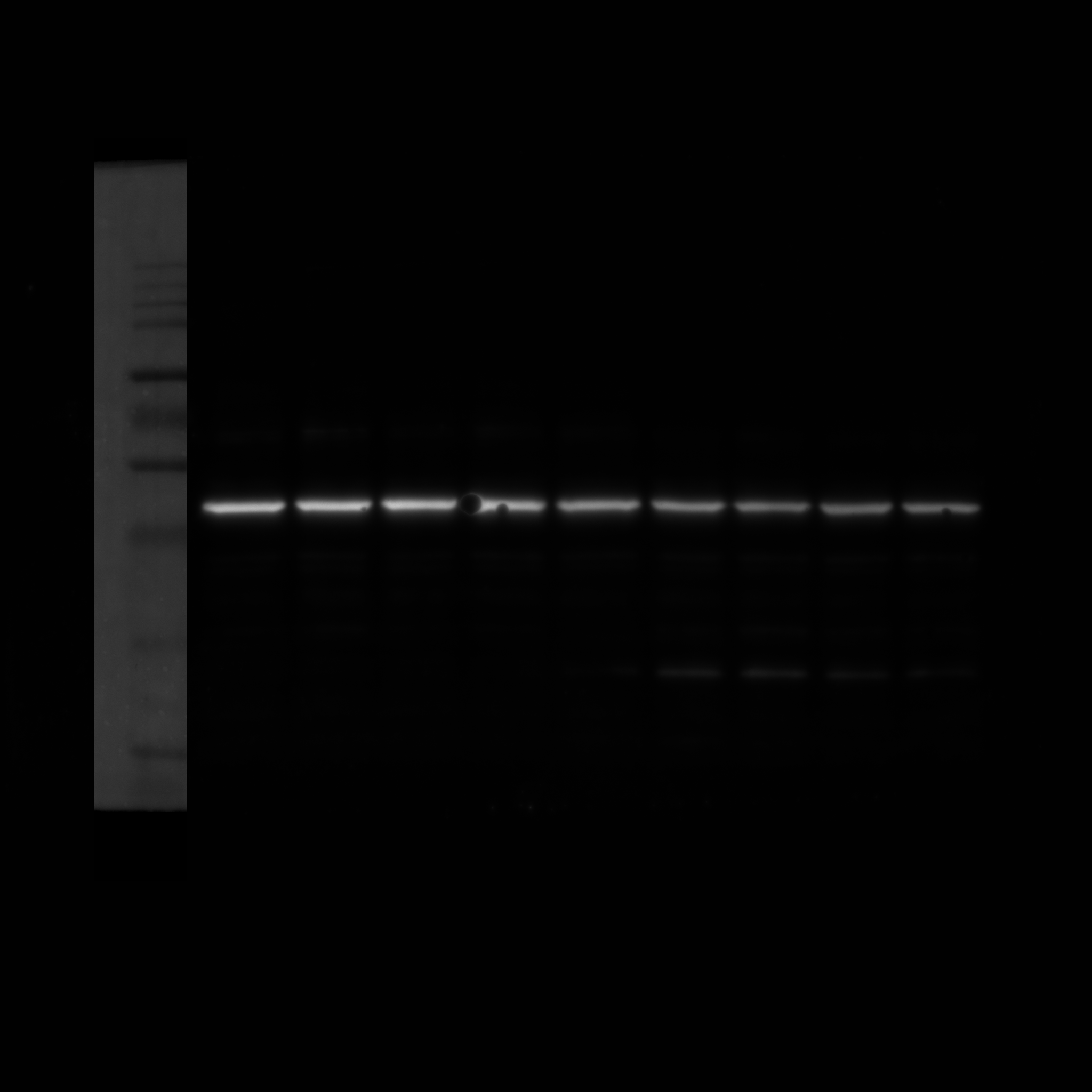

Supplement: S1 Files — Exposures of immunoblot membranes 1, 2, and 3 were used for Fig 2 and exposures of membranes 3, 4 and 5 for S2 Fig. The immunoblot TIFF files are ordered according to stripping steps (1 = before stripping). The positions of the co-electrophoresed size markers were inserted with FusionCapt Advance software version 16.06 on a Fusion-Solo.WL.4M (Vilber Lourmat). The exact details on the ProSieve QuadColor Protein Marker 4.6–300 kDa can be found on the manufacturer’s homepage at http://www.lonza.com/products-services/bio-research/electrophoresis-of-nucleic-acids-and-proteins/protein-electrophoresis/protein-stains-markers/prosieve-protein-colored-and-unstained-markers.aspx. The polypeptides remaining in the SDS-polyacrylamide gels after blotting onto the PVDF membranes were detected with a colloidal staining solution [20 mM CuSO4, 10% (v/v) acetic acid, 45% (v/v) methanol, 0.15% (w/v) Coomassie Brilliant Blue G250 (SERVA Electrophoresis)] and unbound dye was removed by washing in water. Stained proteins were recorded on a Quantum ST4 1100/26MX (Vilber Lourmat) using Quantum-Capt software version 15.12 to estimate transfer efficiency. They are also included as TIFF files. Measurements used for diagrams and statistical analyses in Fig 5A, 5C, 5D, 5E and Fig 6D are deposited in the measurements.xlsx file. Detailed information about secondary antibodies is included in antibodies.pdf. (ZIP) [file pone.0149106.s005.zip › Membrane 5/151220_2_actin_+ruler.Tif]

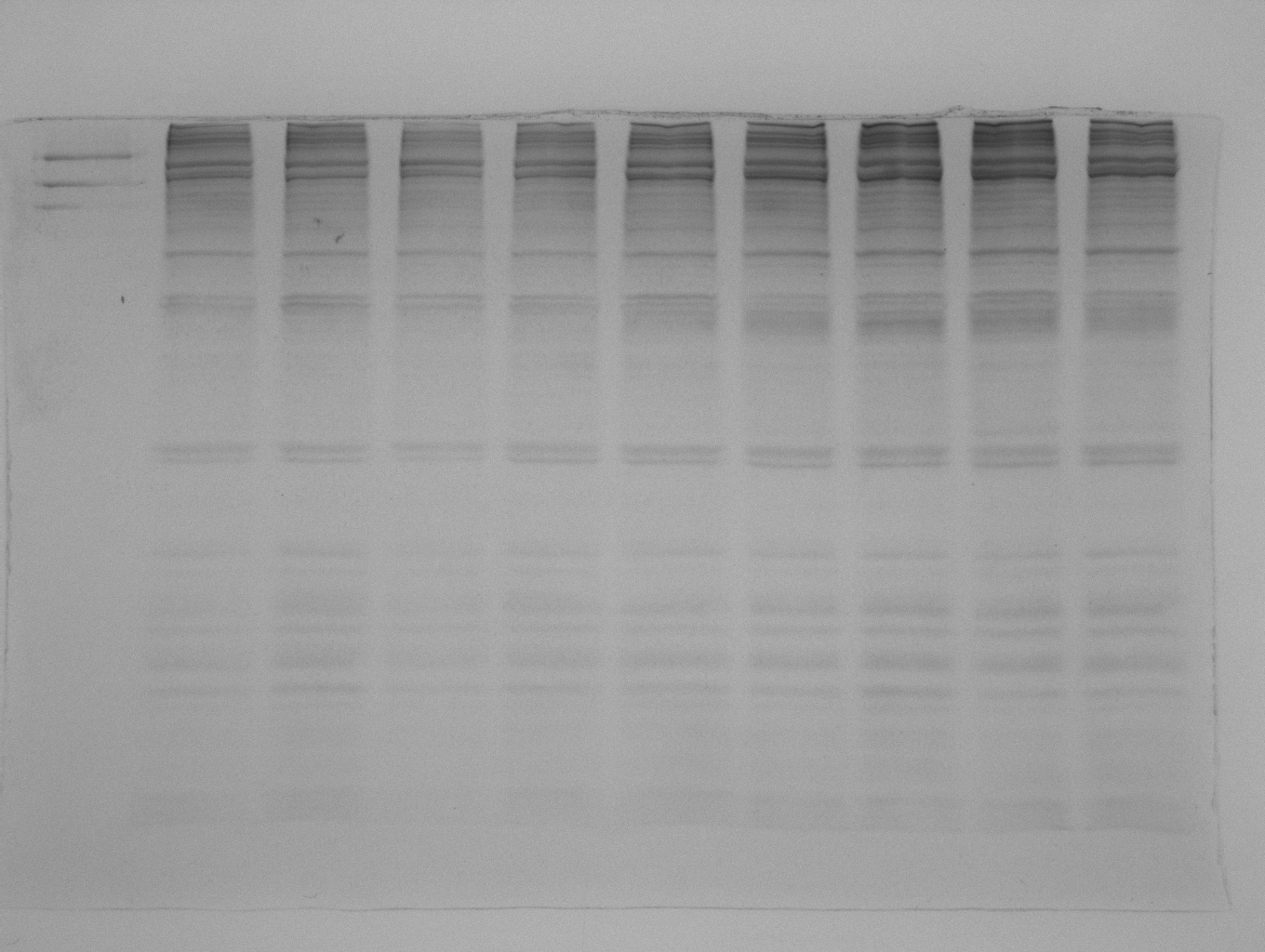

Supplement: S1 Files — Exposures of immunoblot membranes 1, 2, and 3 were used for Fig 2 and exposures of membranes 3, 4 and 5 for S2 Fig. The immunoblot TIFF files are ordered according to stripping steps (1 = before stripping). The positions of the co-electrophoresed size markers were inserted with FusionCapt Advance software version 16.06 on a Fusion-Solo.WL.4M (Vilber Lourmat). The exact details on the ProSieve QuadColor Protein Marker 4.6–300 kDa can be found on the manufacturer’s homepage at http://www.lonza.com/products-services/bio-research/electrophoresis-of-nucleic-acids-and-proteins/protein-electrophoresis/protein-stains-markers/prosieve-protein-colored-and-unstained-markers.aspx. The polypeptides remaining in the SDS-polyacrylamide gels after blotting onto the PVDF membranes were detected with a colloidal staining solution [20 mM CuSO4, 10% (v/v) acetic acid, 45% (v/v) methanol, 0.15% (w/v) Coomassie Brilliant Blue G250 (SERVA Electrophoresis)] and unbound dye was removed by washing in water. Stained proteins were recorded on a Quantum ST4 1100/26MX (Vilber Lourmat) using Quantum-Capt software version 15.12 to estimate transfer efficiency. They are also included as TIFF files. Measurements used for diagrams and statistical analyses in Fig 5A, 5C, 5D, 5E and Fig 6D are deposited in the measurements.xlsx file. Detailed information about secondary antibodies is included in antibodies.pdf. (ZIP) [file pone.0149106.s005.zip › Membrane 5/151220_SDS-PAGE after transfer.Tif]
