# Supplementary material for: Effects of Plectin Depletion on Keratin Network Dynamics and Organization
Source: PLoS One. 2016 Mar 23;11(3):e0149106. doi: 10.1371/journal.pone.0149106 (PMC4805305; doi:10.1371/journal.pone.0149106)
Supplement: S1 Files — Exposures of immunoblot membranes 1, 2, and 3 were used for Fig 2 and exposures of membranes 3, 4 and 5 for S2 Fig. The immunoblot TIFF files are ordered according to stripping steps (1 = before stripping). The positions of the co-electrophoresed size markers were inserted with FusionCapt Advance software version 16.06 on a Fusion-Solo.WL.4M (Vilber Lourmat). The exact details on the ProSieve QuadColor Protein Marker 4.6–300 kDa can be found on the manufacturer’s homepage at http://www.lonza.com/products-services/bio-research/electrophoresis-of-nucleic-acids-and-proteins/protein-electrophoresis/protein-stains-markers/prosieve-protein-colored-and-unstained-markers.aspx. The polypeptides remaining in the SDS-polyacrylamide gels after blotting onto the PVDF membranes were detected with a colloidal staining solution [20 mM CuSO4, 10% (v/v) acetic acid, 45% (v/v) methanol, 0.15% (w/v) Coomassie Brilliant Blue G250 (SERVA Electrophoresis)] and unbound dye was removed by washing in water. Stained proteins were recorded on a Quantum ST4 1100/26MX (Vilber Lourmat) using Quantum-Capt software version 15.12 to estimate transfer efficiency. They are also included as TIFF files. Measurements used for diagrams and statistical analyses in Fig 5A, 5C, 5D, 5E and Fig 6D are deposited in the measurements.xlsx file. Detailed information about secondary antibodies is included in antibodies.pdf. (ZIP) [file pone.0149106.s005.zip › antibodies.pdf]

Fluorophore-conjugated secondary goat antibodies were from Invitrogen.

Fig. 1 and S2 Fig: Alexa Fluor® 488 anti-guinea pig (dilution: 1:1000), Alexa Fluor® 555 anti-rat (1:500), and Alexa Fluor® 647 anti-mouse (1:200).

Fig. 3: Alexa Fluor® 555 anti-guinea pig, and Alexa Fluor® 647 anti-rabbit (1:200).

Fig. 4: Alexa Fluor® 555 anti-rat (1:500), and Alexa Fluor® 647 anti-rabbit (1:200).

S5 Fig: DyLight™ 405 anti-guinea pig (1:150), Alexa Fluor® 555 anti-rat (1:500), and Alexa Fluor® 647 anti-mouse (1:200). Horseradish peroxidase-conjugated secondary goat antibodies were from Dianova and were used at a dilution of 1:5000 for anti-guinea pig, anti-rabbit, and anti-rat, and at a dilution of 1:20000 for anti-mouse.
