# Supplementary material for: Effects of Plectin Depletion on Keratin Network Dynamics and Organization
Source: PLoS One. 2016 Mar 23;11(3):e0149106. doi: 10.1371/journal.pone.0149106 (PMC4805305; doi:10.1371/journal.pone.0149106)
Supplement: S1 Files — Exposures of immunoblot membranes 1, 2, and 3 were used for Fig 2 and exposures of membranes 3, 4 and 5 for S2 Fig. The immunoblot TIFF files are ordered according to stripping steps (1 = before stripping). The positions of the co-electrophoresed size markers were inserted with FusionCapt Advance software version 16.06 on a Fusion-Solo.WL.4M (Vilber Lourmat). The exact details on the ProSieve QuadColor Protein Marker 4.6–300 kDa can be found on the manufacturer’s homepage at http://www.lonza.com/products-services/bio-research/electrophoresis-of-nucleic-acids-and-proteins/protein-electrophoresis/protein-stains-markers/prosieve-protein-colored-and-unstained-markers.aspx. The polypeptides remaining in the SDS-polyacrylamide gels after blotting onto the PVDF membranes were detected with a colloidal staining solution [20 mM CuSO4, 10% (v/v) acetic acid, 45% (v/v) methanol, 0.15% (w/v) Coomassie Brilliant Blue G250 (SERVA Electrophoresis)] and unbound dye was removed by washing in water. Stained proteins were recorded on a Quantum ST4 1100/26MX (Vilber Lourmat) using Quantum-Capt software version 15.12 to estimate transfer efficiency. They are also included as TIFF files. Measurements used for diagrams and statistical analyses in Fig 5A, 5C, 5D, 5E and Fig 6D are deposited in the measurements.xlsx file. Detailed information about secondary antibodies is included in antibodies.pdf. (ZIP) [file pone.0149106.s005.zip › file list.pdf]

List of all files in S1 Files.zip.

antibodies.pdf

measurements.xlsx

Folder Membrane 1:

140213_1_plectin HD1_+ruler.Tif

140213_2_GAPDH_+ruler.Tif

140213_3_plectin_+ruler.Tif

140213_4_integrin b1_+ruler.Tif

140213_5_actin_+ruler.Tif

140213_6_integrin a3_+ruler.Tif

140213_SDS-PAGE after transfer.Tif

Folder Membrane 2:

140211_2_integrin a5_2x binning_+ruler.Tif

140211_3_integrin b5_+ruler.Tif

140211_4_actin_+ruler.Tif

140211_5_integrin b4_+ruler.Tif

140211_SDS-PAGE after transfer.Tif

Folder Membrane 3:

140305_1_integrin a2_2x binning_+ruler.Tif

140305_2_keratin 5_+ruler.Tif

140305_4_actin_+ruler.Tif

140305_SDS-PAGE after transfer.Tif

Folder Membrane 4:

140305_1_keratin 13_binning 2x_+ruler.Tif

140305_2_keratins 8 and 18_+ruler.Tif

140305_3_keratin 8_+ruler.Tif

140305_4_actin_+ruler.Tif

140305_SDS-PAGE after transfer.Tif

Folder Membrane 5:

151220_1_keratin 17_binning 2x_+ruler.Tif

151220_2_actin_+ruler.Tif

151220_SDS-PAGE after transfer.Tif
